# Comparative Transcriptomics and Metabolomics Reveal an Intricate Priming Mechanism Involved in PGPR-Mediated Salt Tolerance in Tomato

**DOI:** 10.3389/fpls.2021.713984

**Published:** 2021-08-17

**Authors:** Ifigeneia Mellidou, Aggeliki Ainalidou, Anastasia Papadopoulou, Kleopatra Leontidou, Savvas Genitsaris, Evangelos Karagiannis, Bram Van de Poel, Katerina Karamanoli

**Affiliations:** ^1^Institute of Plant Breeding and Genetic Resources, Hellenic Agricultural Organization DEMETER (ex NAGREF), Thermi, Greece; ^2^Laboratory of Agricultural Chemistry, School of Agriculture, Aristotle University of Thessaloniki, Thessaloniki, Greece; ^3^Section of Ecology and Taxonomy, School of Biology, National and Kapodistrian University of Athens, Athens, Greece; ^4^Laboratory of Pomology, Department of Horticulture, Aristotle University of Thessaloniki, Thessaloniki, Greece; ^5^Division of Crop Biotechnics, Department of Biosystems, University of Leuven, Leuven, Belgium

**Keywords:** abiotic tolerance, ACC-deaminase, antioxidant metabolism, bio-stimulants, priming state, *Pseudomonas oryzihabitans*

## Abstract

Plant-associated beneficial strains inhabiting plants grown under harsh ecosystems can help them cope with abiotic stress factors by positively influencing plant physiology, development, and environmental adaptation. Previously, we isolated a potential plant growth promoting strain (AXSa06) identified as *Pseudomonas oryzihabitans*, possessing 1-aminocyclopropane-1-carboxylate deaminase activity, producing indole-3-acetic acid and siderophores, as well as solubilizing inorganic phosphorus. In this study, we aimed to further evaluate the effects of AXSa06 seed inoculation on the growth of tomato seedlings under excess salt (200 mM NaCl) by deciphering their transcriptomic and metabolomic profiles. Differences in transcript levels and metabolites following AXSa06 inoculation seem likely to have contributed to the observed difference in salt adaptation of inoculated plants. In particular, inoculations exerted a positive effect on plant growth and photosynthetic parameters, imposing plants to a primed state, at which they were able to respond more robustly to salt stress probably by efficiently activating antioxidant metabolism, by dampening stress signals, by detoxifying Na^+^, as well as by effectively assimilating carbon and nitrogen. The primed state of AXSa06-inoculated plants is supported by the increased leaf lipid peroxidation, ascorbate content, as well as the enhanced activities of antioxidant enzymes, prior to stress treatment. The identified signatory molecules of AXSa06-mediated salt tolerance included the amino acids aspartate, threonine, serine, and glutamate, as well as key genes related to ethylene or abscisic acid homeostasis and perception, and ion antiporters. Our findings represent a promising sustainable solution to improve agricultural production under the forthcoming climate change conditions.

## Introduction

In the forthcoming years, global challenges due to climate change and environmental stresses, including soil salinity, are expected to significantly alter soil properties, causing detrimental effects not only on plant growth and crop production, but also on cultivable area worldwide due to salinization (Liu et al., 2017; Kozminska et al., 2018; Corwin, 2021). Apart from disrupting ion balance and cellular homeostasis, high Na^+^ concentrations in the soil trigger osmotic stress that impairs water usage, photosynthesis, and biosynthesis of proteins and lipids, as well as cell redox state by over-accumulating reactive oxygen species (ROS) (Liu et al., 2017). Excess salt may activate a wide range of physiological and biochemical adjustments to support plant growth and cellular functions. These include the effective compartmentalization of Na^+^ in vacuoles by specific transporters, long-distance ion transport from roots to leaves and stems, alterations in leaf or root morpho-anatomical structures, as well as the production of osmotically active compounds and the accumulation of plant hormones and other signaling molecules (Shabala, 2013; Mellidou et al., 2016; Devkar et al., 2020).

Among the effective strategies to cope with soil salinity, the use of improved management practices in highly saline regions, as well as the implication of conventional or molecular technologies toward the development of salt tolerant crop species are of outmost importance. However, these strategies are often time consuming and cost-demanding. The application of beneficial microbes represents an alternative environmental-friendly strategy to address this issue (Chauhan et al., 2019). The plant rhizosphere represents a delicate and dynamic ecosystem that hosts a variety of microorganisms with diverse and multifaceted functions in plant growth and development, or survival under unfavorable environmental conditions (Kearl et al., 2019; Genitsaris et al., 2020). Plant Growth Promoting Rhizobacteria (PGPR) can provide cross-protection against multiple stress factors by facilitating the growth of their plant symbionts in many different ways (Bruto et al., 2014). Among others, they supply atmospheric nitrogen, synthesize siderophore and phytohormones, solubilize inorganic phosphorus (P), release volatiles, form biofilm, or produce stress alleviating enzymes, such as the 1-aminocyclopropane-1-carboxylate (ACC) deaminase, and cell-wall degrading enzymes (Mayak et al., 2004; Yang et al., 2009; Shariati et al., 2017; Leontidou et al., 2020; Jiao et al., 2021). PGPR-mediated salt tolerance may be achieved via modifications in Na^+^, K^+^, and Ca^+2^ homeostasis thereby allowing plants to sustain a higher K^+^/Na^+^ within cells (Bharti et al., 2016; Safdarian et al., 2019). At the molecular level, salt stress regulates the expression of several genes coding for late embryogenesis (LEA) proteins, osmoregulatory proteins, redox-regulated proteins, transcription factors (TFs) such as WRKY, transporters/antiporters, and salt overly sensitive (SOS) proteins (Safdarian et al., 2019).

In an attempt to investigate plant microbiomes and the co-evolutionary signature of host-microorganism interactions, a large number of emblematic PGPR model strains have been isolated, characterized and applied as “biostimulants” to enhance plant abiotic stress tolerance (Liu et al., 2017; Olanrewaju et al., 2017; Leontidou et al., 2020; Yadav et al., 2020). The physiological, transcriptomic, and metabolomic profiles of PGPR-mediated salt tolerance have been explored in differed crops including *Arabidopsis* (Liu et al., 2017), rice (Chauhan et al., 2019), and wheat (Safdarian et al., 2019), suggesting intricate metabolic and hormonal signaling events implicated in plant responses against salt stress.

Plant-associated beneficial strains inhabiting arid and harsh ecosystems may help plants survive under abiotic and biotic stress factors, as these microbes have developed complex adaptive traits in co-evolution with their plant hosts, in contrast to those found in frequently cultivated areas (Fierer, 2017). Previously, we were able to isolate a potential PGPR strain (AXSa06), identified as *Pseudomonas oryzihabitans*, from the National Park of Delta Axios (Leontidou et al., 2020), a highly diverse ecosystem which includes the estuaries of four rivers and has been included in the Natura 2000 network of European ecological regions with code GR122000268 (Vokou et al., 2016). *In vitro* experiments showed that AXSa06 possessed ACC deaminase activity, produced a considerable amount of IAA and siderophores, as well as solubilized inorganic P (Leontidou et al., 2020). PGPR strains containing ACC deaminase activity may help plants ameliorate stress injury by promoting plant and root growth, through scavenging plant ACC, thereby reducing ethylene production (Orozco-Mosqueda et al., 2020). Although this strain has been isolated from a perennial *Sarcocornia* sp. grown in highly saline soils, it could efficiently colonize the rhizosphere of tomato plants (Leontidou et al., 2020).

Due to its interesting phyto-beneficial traits, we aimed to further evaluate the effects of AXSa06 seed inoculation on the growth of tomato seedlings under excess salt (200 mM NaCl). Deciphering transcriptomic and metabolomic changes of inoculated plants exposed to salt stress provided important insights into the complicated mechanism that allowed our novel strain to induce salt tolerance in plants. The proposed model suggests that AXSa06 forced plants to a “primed” state. This state is related to specific metabolite accumulation, repressed stress-inducing signals through a dampened ethylene and abscisic acid (ABA) metabolism, and eventually in effective trade-off between growth and defense responses. The identified signatory molecules including AsA, and the amino acids aspartate, threonine, serine, and glutamate, may serve as possible biomarkers for PGPR priming in tomato.

## Materials and Methods

### Plant Material, Growth Conditions, and Salt Treatment

Tomato seeds of cv “ACE 55” were used to explore plant responses to salt stress upon AXSa06 inoculation. The strain has been previously isolated from an adverse—highly saline—ecosystem (National Park of Delta Axios, Greece) and genetically characterized as *Pseudomonas oryzihabitans* using a Whole Genome Sequencing approach (Leontidou et al., 2020). Seeds were surface sterilized in 70% ethanol (v/v) and subsequently in 2.4% (v/v) sodium hypochlorite. Prior sterilization, seeds were kept in the dark for 24 h at 4°C and then incubated for 72 h in PNS agar plates (1.8% agar) at 25°C to ensure synchronization in growth.

For inoculation with the selected bacteria, called AXSa06, spontaneous rifampicin-resistant mutants were prepared as described earlier (Karamanoli et al., 2005) in order to easily monitor bacterial dynamics in the rhizosphere during experimentation. Seeds were “bacterized” for 30 min in a mixture of the bacterial suspension and 2% methyl cellulose (MC) solution (w/v) (Sigma, Germany) in a 1:1 (v/v) ratio, as previously described (Leontidou et al., 2020). The control seeds were immersed in a mixture of Phosphate Buffer Saline (PBS) and MC (1:1). In turn, seeds of both treatments were placed in pots filled with sterilized peat and perlite (3:1 v/v) in a growth chamber (Snijders Microclima 1,750, Snijders Scientific BV, Netherlands) for 3 weeks at 16/8 h and 25/22°C, receiving plant nutrient medium containing 0.5% (w/v) sucrose (PNS). Salt stress was applied to 21-day old seedlings by the addition of 200 mM NaCl for 7 days based on our preliminary experiments in tobacco (Mellidou et al., 2016) and tomato (Leontidou et al., 2020). Plants received equal volume of 200 mM NaCl solution or tap water (control plants), without leaching. The concentration of NaCl in the pots was regularly monitored by measuring the Electrical Conductivity (EC) after the completion of the irrigation, and values had to be in the range of 3.0–3.5 dS m^−1^. In total, there were four treatments, (1) non-inoculated non-stressed (0 mM NaCl), (2) non-inoculated salt-stressed (200 mM NaCl), (3) AXSa06-inoculated non-stressed, and (4) AXSa06-inoculated salt-stressed, each consisted of 20 plants (Supplementary Figure 1). At the end of the stress period, plant growth-related and physiological parameters were evaluated, while leaf and root tissues were frozen in liquid nitrogen and kept at −80°C until further analysis.

### Plant Growth and Physiological Measurements

Shoot length and total leaf number were recorded for all plants exposed to 200 mM NaCl for 7 days, while above the ground biomass (stems and leaves), on a fresh weight basis, was evaluated for 10 plants per treatment, at the end of stress treatment. Among the physiological parameters, net photosynthetic rate (A_net_, mmol m^−2^ s^−1^), and chlorophyll content index [CCI = (% transmittance at 931 nm)/(% transmittance at 653 nm)] were assessed in 10 plants per treatment using a portable photosynthesis meter (LCPROT-001/BW, ADC BiosScientific Ltd., UK), and an Opti-Sciences CCM-200 chlorophyll content meter (OptiSciences Inc.), respectively. Measurements were taken on the second fully expanded leaf (counting from the apex) as described earlier (Mellidou et al., 2016, 2017).

### Salt Stress Markers Evaluation

The accumulation of Na^+^ in tomato leaves inoculated with AXSa06 following treatments with 200 mM NaCl for 7 days was determined as previously described (Mellidou et al., 2016).

Briefly, leaf samples (0.5 g) were heated in a muffle furnace at 500°C for at least 4 h, and the residue was dissolved in a 3.6 M HCl/1.4 M HNO_3_ aqueous solution. In turn, Na^+^ content was determined in a flame photometer (Jenway PFP 7, Gransmore Green, Felsted England).

Malonyldialdehyde (MDA) content was determined in leaf samples to estimate the degree of lipid peroxidation using the thiobarbituric acid (TBA) test (Heath and Packer, 1968), with a few modifications (Mellidou et al., 2020). Briefly, frozen leaf powder (200 mg) was homogenized in 600 mL 0.1% (w/v) trichloroacetic acid (TCA) solution, and centrifuged at 14,000 rpm for 15 min at 4°C. In turn, 0.5 mL of the supernatant was added to 1.5 mL 0.5% (w/v) TBA in 20% TCA, the mixture was boiled for 25 min, and the reaction was completed by immersing the reaction tubes on ice. The MDA content was calculated by measuring the absorbance of supernatant at 532 nm, after subtracting the value for non-specific absorption at 600 nm, using an extinction coefficient of 155 mM^−1^ cm^−1^.

REL was determined essentially as previously described (McKay, 1998). Briefly, at the end of stress treatment, roots were thoroughly rinsed with tap water, immersed in glass tubes containing 15 ml of deionized water with known EC and vortexed vigorously. After 24 h in dark, EC was measured before and after boiling at 110°C for 20 min using the electrical conductivity meter ConductoMeter (Metrohm, Herisau, Switzerland). The injury index was estimated from the formula: REL (%) = [(EC before/EC after boiling) × 100]/initial root weight. Measurements were performed using five replicates per treatment.

Proline content was also assayed in leaf tissues according to Bates (1976). Briefly, 100 mg of leaf powder was homogenized in 5 ml of 3% (w/v) aqueous sulfosalicylic acid and then centrifuged at 14,000 rpm for 10 min at 4°C. One ml acid-nynhydrin reagent and 1 ml glacial acetic acid was added to 1 ml of supernatant, and the mixture was heated at 100°C for 1 h. After terminating the reaction in an ice bath, the reaction mixture was extracted with 2 ml toluene, vortexed vigorously, and left at room temperature for 30 min. Proline content was determined by measuring the absorbance of the fraction with toluene at 520 nm, based on a standard curve with pure proline of known concentrations.

For all measurements, five biological replications per treatment were used, corresponding to five individual plants.

### Determination of Non-enzymatic and Enzymatic Antioxidants

Ascorbic acid (AsA) was determined spectrophotometrically using the ascorbate oxidase (AO) enzyme as previously described (Pateraki et al., 2004). Calculations were based upon the difference in absorbance at 265 nm before and 3 min after the addition of AO (1 U/μl) to a 200 μL aliquot of extract in 200 mM sodium phosphate buffer (pH 5.6). Total AsA (totAsA) content was determined by measuring absorbance 10 min after adding 10 mM DTT to a separate extract aliquot. Measurements were performed using five biological and two technical replicates per treatment.

For enzyme activities, total proteins were extracted from 500 mg of leaf tissues, and purified using ion-exclusion Sephadex G-25 column (PD 10, GE Healthcare) essentially as previously described (Mellidou et al., 2012). Bovine serum albumin (Sigma) was used as standard for the quantification of protein content of the extracts according to Bradford method (Bradford, 1976). The activities of catalase (EC 1.11.1.6, CAT), superoxide dismutase (SOD; EC 1.15.1.1), pyrogallol-based peroxidase (EC 1.11.1.7, POX), and ascorbate peroxidase (EC 1.11.1.11, APX) were determined based on optimized “in-house” protocols (Mellidou et al., 2012, 2017). All technical measurements were conducted in duplicates for each of the five biological replicates per treatment.

### Quantification of ACC and MACC Content in Leaves and Roots

As AXSa06 strain possesses ACC deaminase gene, as well as *in vitro* ACC activity (Leontidou et al., 2020), metabolites from the ethylene biosynthetic pathway (ACC and MACC) were quantified in leaves and roots of the plants at exposure to 200 mM NaCl, essentially as previously described (Bulens et al., 2011; Van de Poel et al., 2014). Enzyme activities of ACC synthase and ACC oxidase were below the threshold of detection limit, and thus not able to be quantified.

### Ethylene Quantification in Excised Tomato Leaves

Ethylene production was quantified in excised non-inoculated and AXSa06-inoculated tomato leaves upon salt stress as previously described (Kim et al., 2013, 2016). Briefly, leaves were excised, weighted, and placed in a glass tube. After 30 min without sealing, the glass tubes were capped with a septa stopper, and incubated for an hour at room temperature. One ml gas sample was removed from the tube using a syringe and injected into a gas chromatograph (GC-2014ATF Shimadzu), equipped with a flame ionization detector (FID) and a stainless-steel column (filled with Porapak P, Q, R, S), to quantify ethylene gas levels. Measurements were performed using five individual biological replicates per treatment.

### RNA Isolation, Library Construction, and Sequencing

Leaf tissues were used for total RNA extraction using RNeasy Plant Mini Kit (QIAGEN) according to the manufacturer's instructions. Three biological replicates were employed per treatment. Quantification and qualification of the extracted RNA was checked using the RNA Nano 6000 Assay Kit of the Agilent Bioanalyzer 2100 system (Agilent, CA, USA) to ensure that the RNA Integrity Number (RIN) values were above 7. In total, 12 RNA-Seq libraries were prepared and purified using QIAseq Stranded mRNA Select Kit (QIAGEN) following manufacturer's instructions. After generating the index-coded samples, each cDNA library was sequenced in a single lane of Illumina platforms (Novaseq 6000 System) with 150 bp paired-end reads, obtaining above 24 M raw reads per sample. Raw data obtained from sequencing were converted to Fastq format and deposited to the European Nucleotide Archive, under PRJEB42497.

### Analysis of Sequencing Data

Raw sequences were processed with fastp (Chen et al., 2018) to remove reads containing adapter bases, poly-N sequences, and reads with low quality. After quality control, high quality paired-end reads were aligned to the tomato reference genome (Hosmani et al., 2019) using HISAT2 (Kim et al., 2015). The Stringtie (Kovaka et al., 2019) was used to assemble the set of transcript isoforms obtained in the mapping step, and gffcompare (Pertea and Pertea, 2020) was used to compare the assemblies to reference annotation files and sort out new genes from known ones. The read counts mapped for each gene, including known and novel genes, were produced using Featurecounts (Liao et al., 2014), and fragments per kilobase for exon model (FPKM) was calculated for each gene. Differential gene expression levels between treatments (consisted of three biological replicates) were determined using DESeq2 R package. *P*-values were corrected with the Benjamini-Hochberg adjustment within the R *p*.adjust function and the FDR (False Discovery Rate) was also calculated among the genes. Genes adjusted *P* ≤ 0.01 and log_2_ (fold-change) ≥1.5 were considered as up-regulated, and those with log_2_ (fold-change) ≤ 1.5 as down-regulated. Gene ontology (GO) and KEGG enrichment analysis of DEGs was accomplished using clusterProfiler (Yu et al., 2012).

### Metabolite Extraction and Derivatization

Analysis of polar metabolites was performed as previously described (Ainalidou et al., 2016). Leaf tissues (500 mg) were homogenized in liquid nitrogen and transferred to 2 mL screw cap tubes with 1,400 μL of 100% methanol. Adonitol (100 μL of 0.2 mg mL^−1^ aqueous solution) was added as internal quantitative standard and vortexed, following incubation at 70°C for 10 min. Samples were then centrifuged at 11,000 × g (4°C) for 10 min and the supernatants were transferred to glass vials. Subsequently, 750 μL of chloroform and 1,500 μLl of cold distilled H_2_O were added. The mixtures were centrifuged at 2,200 g (4°C) for 15 min and 150 μL from the upper phase (polar phase) were transferred into new 1.5 mL glass vials. The vials were placed in a vacuum desiccator for overnight drying. For the derivatization, dried residues were re-dissolved in 40 μL of 20 mg mL^−1^ methoxyamine hydrochloride in pyridine, and were shaken at 37°C for 2 h. For the completion of derivatization, samples were treating with 70 μL of N-Methyl-N-(trimethylsilyl)-trifluoroacetamide (MSTFA) reagent and incubated at 37°C for 30 min. The aliquots were stored at −20°C, until further analysis by a GC–MS system.

### Gas Chromatography–Mass Spectrometry (GC–MS)–Based Metabolite Profiling

GC–MS analysis was performed in Thermo Trace Ultra GC equipped with ISQ MS and TriPlus RSH autosampler (Switzerland). One microliter sample volumes were injected with a split ratio of 70:1. Separations were carried out on a TR-5MS capillary column 30 m × 0.25 mm × 0.25 μm. Injector temperature was set at 220°C, ion source at 230°C, and the interface at 250°C, while a constant flow rate of the carrier gas (He) was set at 1 mL min^−1^. The GC temperature program was held at 70°C for 5 min, then increased to 240°C at a rate of 8°C min^−1^, and held at 240°C for 15 min. After 5 min solvent delay, mass range of m/z 50–600 was recorded. The mass spectra were acquired in electron impact ionization (EI) mode. The peak area integration and chromatogram visualization were performed using Xcalibur processing program. For peak identification and mass spectra tick evaluation was performed and NIST11 database were used. Mass spectra were cross referenced with those of authentic standards in the GOLM metabolome database (Kopka et al., 2005; Ainalidou et al., 2016). Quantification of the detected metabolites was assessed based on the relative response compared to adonitol as the internal standard, and expressed as relative abundance.

### Statistical Analysis

All data were analyzed by SPSS (Version 25. Chicago, SPSS Inc.). Duncan multiple-range tests were performed to determine significant differences between inoculated vs. non-inoculated seedlings under control or salt stress conditions.

## Results

### AXSa06 Inoculation Improved Morpho-Physiological and Biochemical Traits in Tomato During Salt Stress

**Figure 1:**
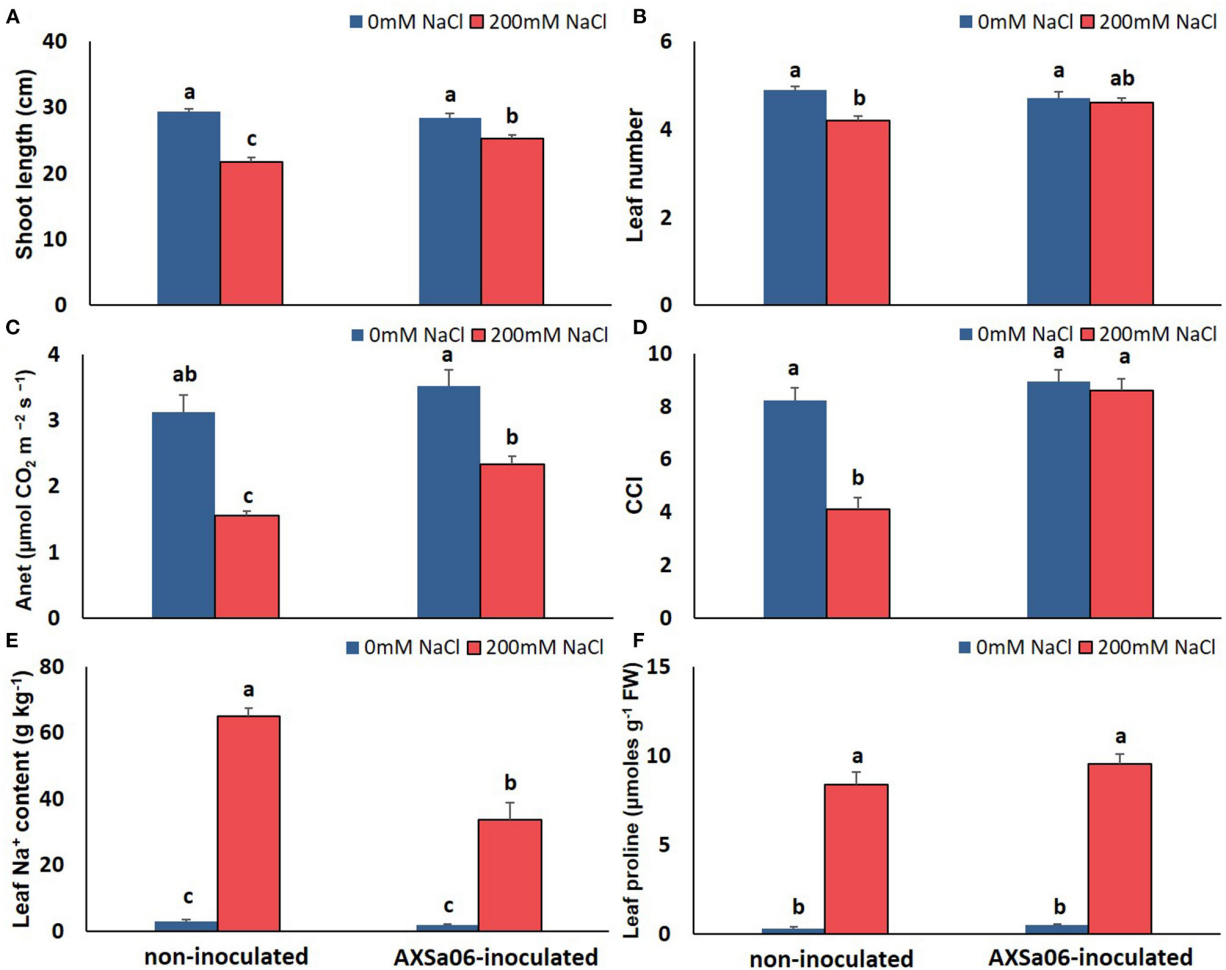
Effect of AXSa06 (*Pseudomonas oryzihabitans*) seed inoculation on tomato seedlings treated with 200 mM NaCl for 7 days: **(A)** shoot length (cm); **(B)** leaf number; **(C)** net photosynthesis (Anet, μmol CO_2_ m^−2^ s^−1^); **(D)** chlorophyll content index; **(E)** leaf Na^+^ content (g kg^−1^); **(F)** leaf proline content (μmols g^−1^ FW). Data are means ± standard error. Different letters indicate statistically significant differences between treatments based on Duncan's multiple range test (*P* < 0.05).

In order to easily monitor strain population throughout the experiment, rifampicin resistant mutants were employed. Under the selected experimental growth conditions, the AXSa06 strain was well-established in the rhizosphere of inoculated plants prior to stress application, while it was also able to grow at highly saline soil conditions (Leontidou et al., 2020). To confirm that AXSa06, identified as *Pseudomonas oryzihabitans*, was able to alleviate salt stress in tomato seedlings, plant-growth, and physiological traits were recorded in treatments with or without 200 mM NaCl. At control conditions (0 mM NaCl), no particular differences related to growth or photosynthesis were recorded between non-inoculated and AXSa06-inoculated plants (Figures 1A–D). Treatments with 200 mM NaCl led to a significant decrease in both shoot length (Figure 1A) and leaf number (Figure 1B) only in non-inoculated plants compared to non-stressed plants. Inoculated plants, not only maintained their growth-related traits at non-stressed levels, but also had higher shoot length (14.2%; Figure 1A) and more leaves (8.7%; Figure 1B) compared to non-inoculated stressed plants. Salt stress caused a significant decrease in A_net_ (50.5% Figure 1C) and CCI (50.2%; Figure 1D), respectively, in non-inoculated plants compared to control conditions. By contrast, the negative effect of 200 mM NaCl in AXSa06-inoculated plants was less prominent, with a 33.9 and 3.9% reduction in A_net_ and CCI, respectively. Both A_net_ and CCI were higher in inoculated plants compared to non-inoculated ones (33.4 and 52.3%, respectively) under salt stress. These results support the positive effect of inoculation with AXSa06 on plant growth and physiological parameters of tomato seedlings exposed to 200 mM NaCl.

**Figure 2:**
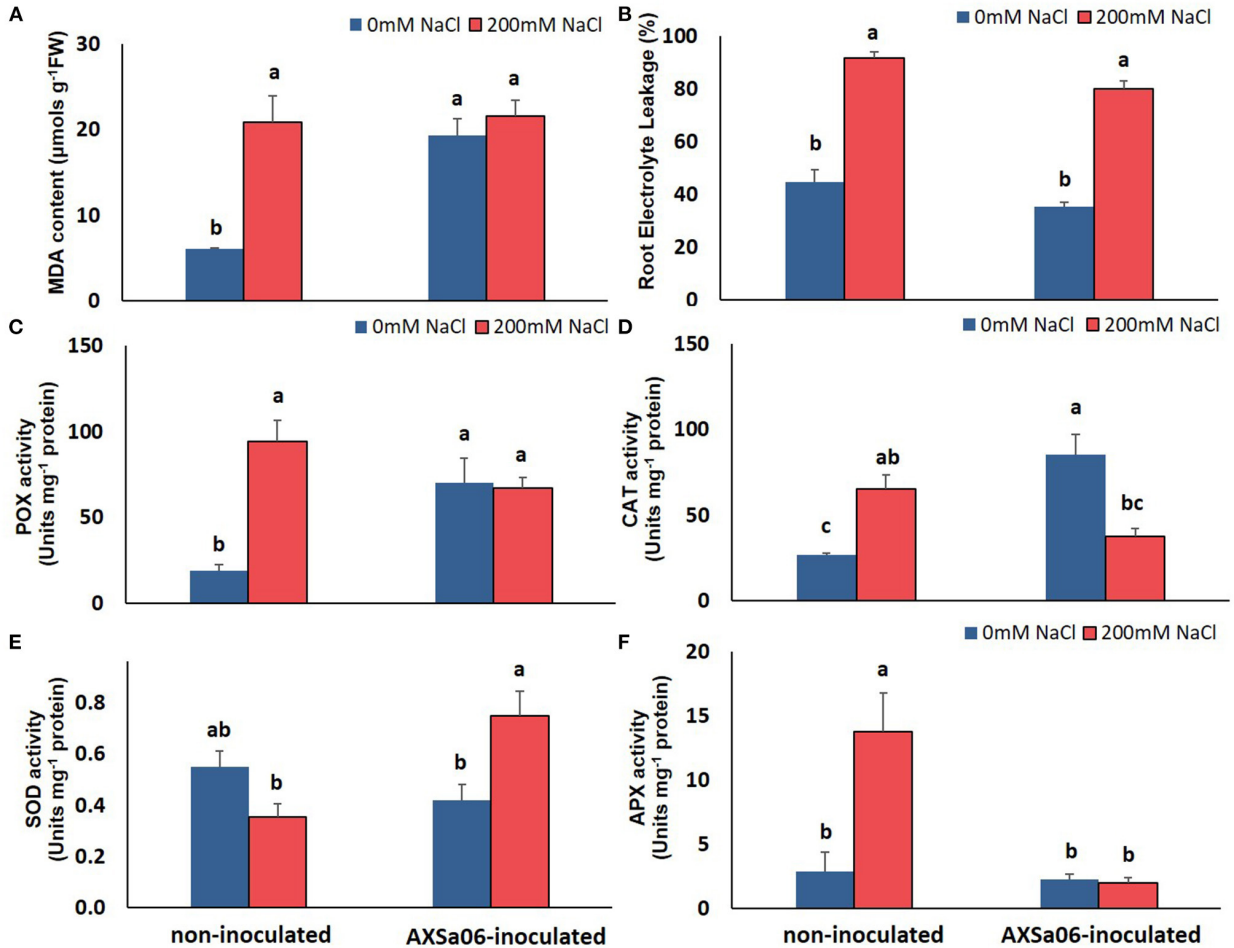
Oxidative-related effect of AXSa06 (*Pseudomonas oryzihabitans*) seed inoculation on tomato seedlings treated with 200 mM NaCl for 7 days: **(A)** MDA content (μmols g^−1^ FW); **(B)** Root Electrolyte Leakage (%); **(C)** POX specific activity (Units mg^−1^ protein); **(D)** CAT specific activity (Units mg^−1^ protein); **(E)** SOD specific activity (Units mg^−1^ protein); **(F)** APX specific activity (Units mg^−1^ protein). Data are means of five biological replicates ± standard error. Different letters indicate statistically significant differences between treatments based on Duncan's multiple range test (*P* < 0.05).

The accumulation of leaf Na^+^ and proline contents was dramatically enhanced at exposure of plants to 200 mM NaCl (Figures 1E,F). Nonetheless, leaves of non-inoculated plants contained significantly higher amount (54.0%) of Na^+^ compared to inoculated ones, while inoculated plants accumulated slightly more proline (12.4%) in leaves than non-inoculated ones, yet not significant. Interestingly, in the absence of NaCl, inoculation with AXSa06 resulted in enhanced leaf MDA content (68.6% higher compared to non-inoculated plants), suggesting that inoculation with the strain probably imitated the effect of mild oxidative stress (Figure 2A). With regard to REL, it was significantly enhanced at exposure to 200 mM NaCl, regardless the presence of AXSa06 (Figure 2B). Nonetheless, REL of AXSa06-inoculated plants at exposure to salt stress was lower (80.23% on average) compared to non-inoculated plants (91.83%), although not significant due to the large scale of values, indicating that there was probably less negative impact of salt in the roots at the presence of the strain. At the same time, the fact that REL of unstressed inoculated plants was also lower (35.5%) compared to non-inoculated plants (44.6%) suggested that the potential oxidative boost at control conditions, as highlighted by the enhanced MDA content, was not harmful with regard to electrolyte leakage from roots.

This mild oxidative boost in AXSa06-inoculated plants was further supported by the increased activities of antioxidant enzymes, POX (Figure 2C) and CAT (Figure 2D), at control conditions. In particular, POX and CAT specific activities were 73.0 or 68.4% higher in inoculated plants compared to non-inoculated ones, respectively. When 200 mM NaCl was applied, no further induction in MDA content or POX activity was observed in inoculated plants, likewise in non-inoculated plants, in which both traits were remarkably enhanced. By contrast, CAT activity decreased in inoculated plants at exposure to salt stress, nearly at control levels of non-inoculated plants. Upon salt stress, SOD activity was significantly increased in inoculated plants, but remained unaltered in non-inoculated ones (Figure 2E). The activity of APX, the first enzyme in the AsA-GSH (glutathione) cycle responsible for the oxidation of AsA to monodehydroascorbate (MDHA) with the parallel detoxification of H_2_O_2_, was increased in non-inoculated plants at exposure to stress. Notwithstanding, APX activity remained at low levels in inoculated plants (Figure 2F), probable due to the lower H_2_O_2_ accumulation within the cell owing to its better detoxification by other peroxidases (Figure 2C).

### Inoculation With AXSa06 Altered Root and Leaf ACC Levels Under Salinity Stress

**Figure 3:**
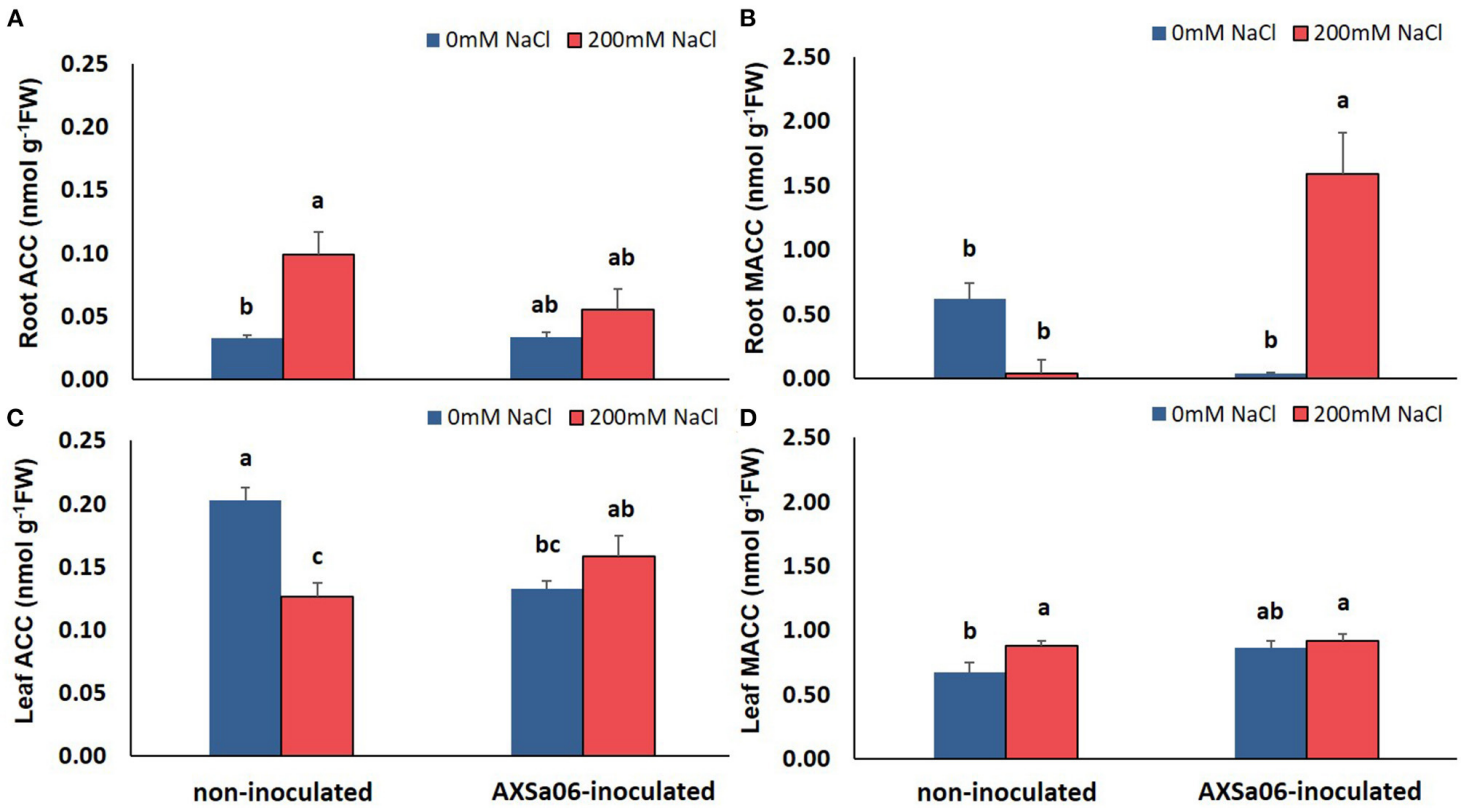
Contents of ACC and MACC in roots **(A,B)** and leaves **(C,D)** of tomato seedlings inoculated or not with AXSa06 (*Pseudomonas oryzihabitans*) at exposure to 200 mM NaCl for 7 days. Data are means of five biological replicates ± standard error. Different letters indicate statistically significant differences between treatments based on Duncan's multiple range test (*P* < 0.05).

In this study, the application of 200 mM NaCl enhanced ACC and reduced MACC levels in the roots of non-inoculated plants (Figures 3A,B). By contrast, inoculation with the ACC-deaminase possessing strain, AXSa06, maintained root ACC content under salt stress similar to control levels, probably due to the ACC deaminase activity of the strain. Intriguingly though, a remarkable increase in root MACC content was observed after inoculation owing to salt stress, probably indicative of a AXSa06-mediated feedback regulation on redirecting plant ethylene synthesis. The opposite trend was evident in the leaves of salt-stressed plants (Figures 3C,D). In particular, at the absence of inoculum, leaf ACC content decreased, probably to support the ethylene production owing to stress, as indicated in the excised leaves of the same plants (Supplementary Figure 2). However, in inoculated plants, ACC levels remained unaltered upon salt stress (Figure 3C), whilst a significant decrease in ethylene production was evident in excised leaves at exposure to 200 mM NaCl (Supplementary Figure 2). Although no *in vivo* ethylene measurements were performed, ethylene determination in excised leaves of plants inoculated with AXSa06 highlighted the capacity of these plants to produce ethylene upon salt stress. Leaf MACC levels remained largely unchanged by both the salinity or inoculation treatment (Figure 3D). These observations suggested that at the presence of AXSa06, the above-the-ground tissues may accumulate lower ACC levels, that could lead to reduced ethylene-related stress signals as a result of ACC deaminase activity below the ground, compared to non-inoculated plants.

### Overview of Transcriptome Analysis and Read Assembly

To unravel the mechanism of AXSa06-mediated salt tolerance in tomato plants, transcriptome profiling was carried out using an RNA-Seq approach. After removing adapter sequences and low-quality reads, a total of 677.6 M with an average of 56.5 M clean reads and a Q30 percentage ≥95% were generated for the 12 cDNA libraries (Supplementary Table 1). No remarkable differences were observed in read number between inoculated vs. non-inoculated plants, or between control and salt stress conditions. The clean reads were mapped to the tomato reference genome, cv Heinz 1706 version 4.0 (Hosmani et al., 2019), with an average total mapping ratio around 97%, and uniquely mapping ratio at 94.6%. After averaging replicate values, total transcripts with FPKM >1 for each treatment varied from 17,873 to 18,153, of which the novel transcripts ranged from 249 to 274 (Supplementary Tables 2–5).

**Figure 4:**
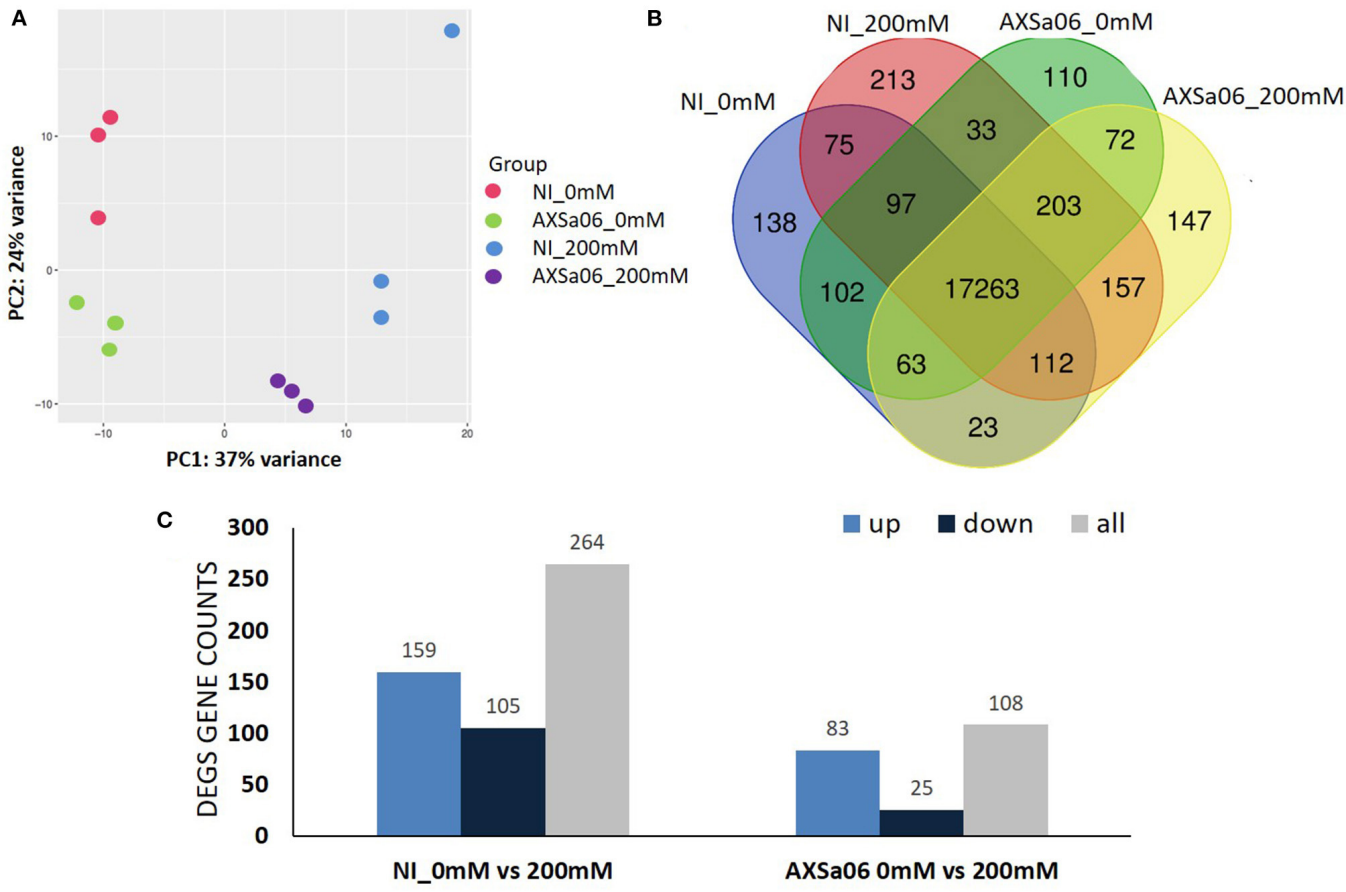
**(A)** Principal Component analysis of all the expressed transcripts with FPKM >1 in non-inoculated (NI) and AXSa06-inoculated plants treated with 0 mM or 200 mM NaCl. **(B)** Venn diagram of the expressed transcripts with FPKM >1 highlighting commonly and uniquely expressed genes between different treatments. **(C)** The number of Differentially Expressed Genes (DEGs) representing up- and down-regulated transcripts in non-inoculated and inoculated plants due to NaCl treatment.

The expression of these transcripts was subjected to a Principal component analysis (PCA) (Figure 4A). The first two components, which explained 61% of the variation, were able to distinguish the four different groups of treatments, with three biological replicates clustering together, and each group having certain differences in their gene expression profiles. Furthermore, at total number of 17,263 transcripts (on average 96% of total expressed genes) were expressed in all treatments, with the uniquely expressed transcripts ranging from 110 (AXSa06-inoculated at 0 mM) to 213 (non-inoculated at 200 mM) (Figure 4B). Differentially Expressed Genes (DEGs) between treatments were defined using |log_2_(fold change) > 1.5|, and corrected (*p* ≤ 0.01). In total, 264 and 108 DEGs were identified between control and salt stress, in non-inoculated or inoculated plants, respectively (Figure 4C and Supplementary Tables 6, 7). Among them, 159 and 83 genes were significantly up-regulated in salt-treated plants in non-inoculated and inoculated plants, respectively, whilst 105 and 25 were down-regulated, suggesting that transcriptomic profiles of non-inoculated plants were more disturbed by NaCl, compared to those of inoculated plants. Although gene expression profiles altered significantly as a response to salt stress to a different extent, there were 43 overlapping DEGs that were regulated by salt stress, regardless inoculations with AXSa06. Among them, the most interesting DEGs up-regulated due to stress involved in central metabolic pathways were alcohol dehydrogenase (ID544074), chitinase (ID544149), _L_-ascorbate oxidase (ID101252861), and proline dehydrogenase (ID778202). In the same regard, 3-ketoacyl-CoA synthase 11 (ID101268510), indole-3-acetic acid-amino synthetase (ID101258277), inositol 2-dehydrogenase (ID101260461), and indole-3-pyruvate monooxygenase YUCCA5 (ID101267265) were significantly down-regulated in both non-inoculated and inoculated plants owing to NaCl treatment. By contrast, there were also two inositol oxygenase genes (ID 101263222 and 101254427) that were up-regulated in inoculated plants but down-regulated in non-inoculated plants.

Gene ontology (GO) annotation analysis of DEGs between stressed and non-stressed plants returned 170 GO terms, classified into the two GO classes (BP, Biological Process; MF, Molecular Function). Among them, 20 or 30 significant GO terms were enriched for non-inoculated and AXSa06-inoculated plants, respectively (Supplementary Table 8 and Supplementary Figure 3). At the absence of inoculum, the most significant (corrected *p* <0.05) enrichment terms of up-regulated genes due to NaCl treatments in the BP category were response to wounding (GO:0009611), and amine metabolic process (GO:0009308), whilst in the MF, there were endopeptidase inhibitor (GO:0004866) and regulator (GO:0061135) activity, peptidase regulator (GO:0061134), and inhibitor (GO:0030414), as well as enzyme inhibitor (GO:0004857), and regulator (GO:0030234) activity (Supplementary Figure 3A). By contrast, at inoculations with AXSa06, GO terms of the BP category such as response to stress (GO:0006950), defense response (GO:0006952), and response to biotic stimulus (GO:0009607) were significantly enriched under salt stress (Supplementary Figure 3B). Among the most significant down-regulated GO terms at the absence of inoculum, there were small molecular metabolic process (GO:0006950), cellular carbohydrate metabolic process (GO:0044262), single organism carbohydrate metabolic (GO:0044723), and catabolic (GO:0044724) processes (Supplementary Figure 3C). Interestingly, the majority of up-regulated GO terms significantly enriched in non-inoculated plants exposed to stress, were identified as significantly down-regulated in inoculated plants (Supplementary Figures 3A,D), suggesting that *Pseudomonas oryzihabitans* AXSa06 strain was able to reverse—to some extent—the modifications stimulated by salt treatment.

As a further step, the DEGs were also examined against KEGG database to identify active biological pathways in tomato seedlings at exposure to salt and AXSa06 treatments (Supplementary Figure 4 and Supplementary Table 9). Assignments of significant DEGs between stressed and non-stressed plants into KEGG pathways showed that the most significant induced pathways were protein processing in endoplasmic reticulum (sly04141), phenylpropanoid biosynthesis (sly00940), plant-pathogen interaction (sly04626), and arginine-proline metabolism (sly00330) in non-inoculated plants (Supplementary Figure 4A), while in AXSa06-plants, pathways of phenylpropanoid biosynthesis (sly00940), as well as starch and sucrose metabolism (sly00500) were up-regulated (Supplementary Figure 4B). The phenylpropanoid metabolic pathway that was induced regardless the presence of the PGP strain, contributes to lignin biosynthesis, including several lignin-forming peroxidases and cell-wall related proteins that were found to be over-expressed upon salt treatment (Supplementary Tables 6, 7). Plant hormone signal transduction pathway (sly04075) was significant down-regulated as a response to stress, regardless inoculation with AXSa06 (Supplementary Figures 4C,D), but in a very different way, as it will be discussed below. By contrast, photosynthetic-related pathways (sly00195 and sly00196), as well as those related to carbon (sly00710, sly 01200), glyoxylate and dicarboxylate (sly00630), or nitrogen metabolism (sly00910), were only repressed at the absence of inoculum in response to salt stress (Supplementary Figure 4B), indicating the notable salt-mediated handicap in primary metabolism. Intriguingly, amino acid biosynthesis (sly01230) was significantly down-regulated in AXSa06-inoculated plants exposed to 200 mM NaCl.

**Figure 5:**
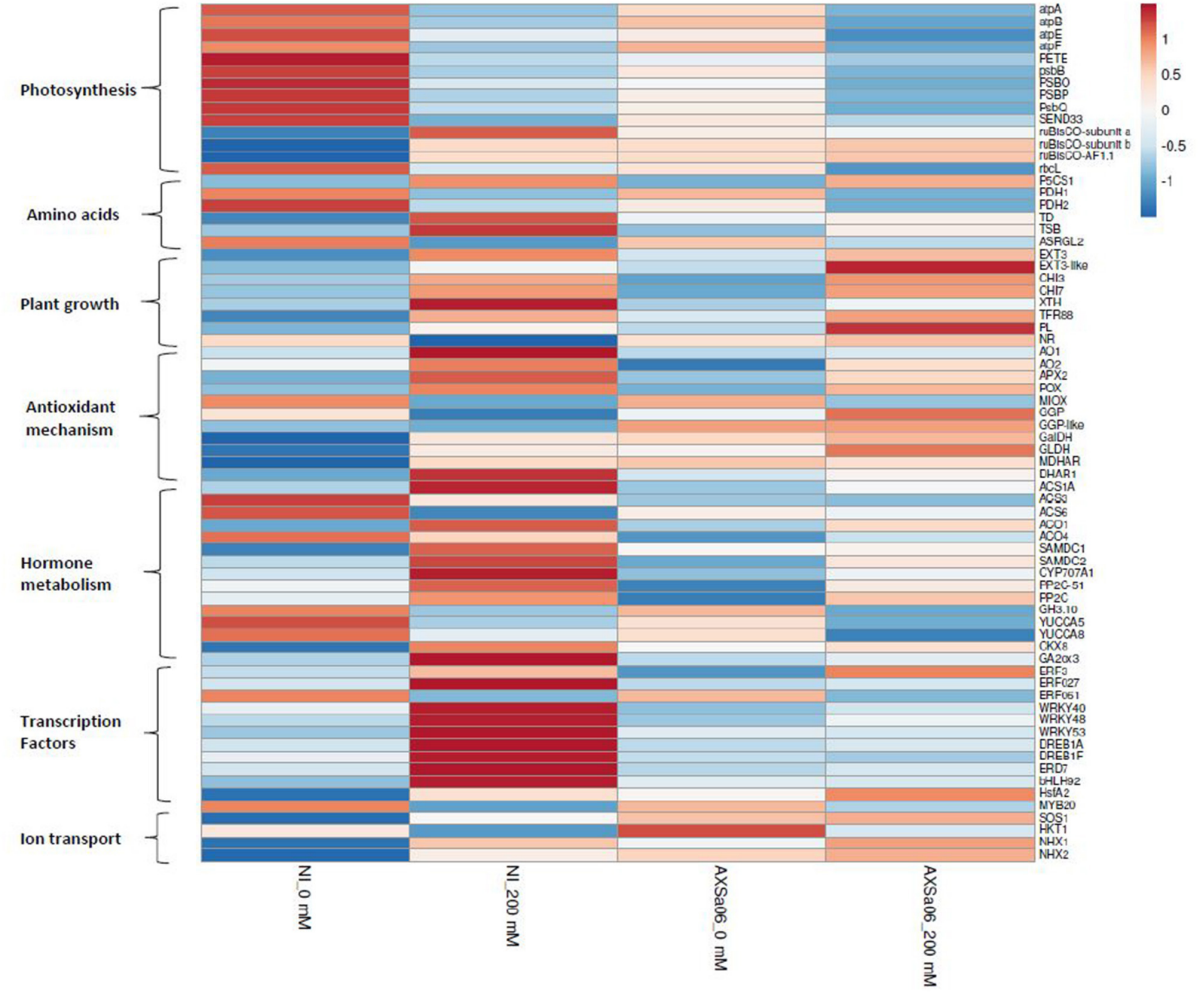
Heatmap of selected genes of interest related to photosynthesis, amino acids, plant growth, antioxidant mechanism, hormone metabolism, transcription factors and ion transport, in the leaves of tomato seedlings inoculated with AXSa06 (*Pseudomonas oryzihabitans*) at exposure to 200 mM NaCl, based on log_2_ transformed FPKM values. The entire list of these genes followed by FPKM values is provided in Supplementary Table 10. NI, non-inoculated.

Transcript abundance of DEGs in non-inoculated and inoculated plants in response to 200 mM NaCl, is also presented using heat maps (Supplementary Figures 5, 6), highlighting a series of changes in gene expression profiles when plants subjected to stress, whilst the entire list of DEGs between stressed and non-stressed plants is also displayed (Supplementary Tables 6, 7). The highest up-regulated genes in non-inoculated plants as a response to salt stress were ferredoxin (ID101248335) and a small heat shock protein (ID544205), whilst the top down-regulated DEGs included a spermidine hydroxycinnamoyl transferase-like (ID101254821), and the MYB transcription factor RADIALIS-like 6 protein (ID101244580) (Supplementary Table 6). By contrast, among the highest up-regulated genes in AXSa06-inoculated plants as a response to salt stress, there were abscisic acid 8'-hydroxylase 3 (ID101254720), and a non-specific lipid-transfer protein 1-like (ID101265675), while among the highest down-regulated genes, there were the MYB transcription factor RADIALIS-like 3 (ID101246530), and gamma-glutamyl peptidase 3-like (ID101254399) (Supplementary Table 7). Below, we explored manually a broad number of genes of particular interest that were modulated by AXSa06-inoculation by focusing on transcripts related to photosynthesis, amino acid metabolism, plant growth and development, stress responses, hormone synthesis and signaling, transcription factors, and other salt-responsive proteins (Figure 5). Genes with FPKM >1 in at least one treatment were considered as expressed (Supplementary Table 10).

#### Energy Metabolism

Some genes of the central metabolism such as phosphoenolpyruvate carboxylase (ID101245149) and O-acyltransferase WSD1 (ID101246264) were significantly up-regulated, and some others including 3-ketoacyl-CoA synthase 11 (ID101268510) were down-regulated, in non-inoculated plants as a response to salt stress (Supplementary Table 6). Others related to lipid-metabolism were down-regulated when non-inoculated plants were exposed to 200 mM NaCl. These included glycerophosphodiester phosphodiesterase (ID101261081), palmitoyl-monogalactosyldiacylglycerol delta-7 desaturase (ID101250586), GDSL esterase/lipase 1 (ID101268445), and 3-ketodihydrosphingosine reductase (ID101248253). Similarly, the expression of several genes involved in these pathways were also induced [O-acyltransferase WSD1 (ID101251623) and GDSL esterase/lipase (ID101250696)] or repressed [glycerophosphodiester phosphodiesterase (ID101261081), 3-ketoacyl-CoA synthase 11 (ID101268510)] under salt condition in AXSa06-inoculated plants (Supplementary Table 7).

Compared to 0 mM NaCl, a wide number of genes related to light reaction in photosynthesis and ATP synthesis were found to be down-regulated upon salt stress in the absence of inoculum (Figure 5 and Supplementary Table 10), as further suggested by KEGG enrichment (Supplementary Table 9). These included the ATP synthases CF1 alpha (*atpA*), beta (*atpB*), epsilon (*atpE*), and CF0 (*atpF*), chloroplastic plastocyanin (*PETE*), photosystem II 23 protein (*PSBP*), photosystem II CP47 apoprotein (*psbB*), chloroplastic oxygen-evolving enhancer protein 1 (*PSBO*), photosystem II oxygen-evolving complex protein 3 (*PsbQ*), and ferredoxin-I (SEND33) (Figure 5). Down-regulation of the above-mentioned photosynthesis-related genes occurred to a lesser extent in AXSa06-inoculated plants upon salt stress, suggesting that the energy metabolism related to photosynthetic apparatus was less disturbed compared to non-inoculated plants. This is in line with the enhanced net photosynthesis in inoculated plants compared to non-inoculated plants at exposure to 200 mM NaCl (Figures 1C,D). Interestingly, transcript levels of the chloroplastic *ruBisCO* large subunit-binding protein subunit alpha (ID101265242), subunit beta (ID101253117) and accumulation factor 1.1 (ID101251433) were also higher in inoculated plants compared to non-inoculated ones at control conditions, suggesting a probable better carbon assimilation due to the inoculum.

#### Amino Acid Metabolism

Several pathways related to the metabolism of amino acids were also modulated in response to salt stress regardless inoculation with AXSa06 (Figure 5 and Supplementary Table 10). Proline is an important osmoprotectant synthesized in plant cells to alleviate salt stress conditions, over-accumulated in stressed plants regardless inoculation with AXSa06 (Figure 1E). In line with this observation, the key gene in the proline biosynthesis, delta-1-pyrroline-5-carboxylate synthase (*P5CS1*; ID101244293), was significantly up-regulated in the presence of salt, probably to help plants survive unfavorable abiotic conditions. Furthermore, the negative regulation of proline dehydrogenase (*PDH*), the enzyme that converts _L_-proline to _L_-glutamate, was conductive to the accumulation of proline in both non-inoculated (ID778202, ID101268445) and inoculated (ID778202) plants in response to NaCl treatment. Intriguingly, at the absence of inoculum, threonine dehydratase (*TD*; ID543983) responsible for the synthesis of _L_-isoleucine from _L_-threonine, and tryptophan synthase beta chain (*TSB*; ID101256422), responsible for the synthesis of _L−_tryptophan from indole and _L_-serine, were significantly up-regulated in stressed plants. By contrast, isoaspartyl peptidase/L-asparaginase 2 (*ASRGL2*; ID101245157) from the asparagine catabolic pathway was down-regulated.

#### Plant Growth and Development

The transcript levels of several genes involved in plant growth-related processes were also induced at 200 mM NaCl, regardless PGPR inoculation. These included extensins (*EXT*; ID544158, ID101249726) and chitinases (*CHI*; ID544149, ID544147) (Figure 5 and Supplementary Table 10). In addition, cell-wall modification enzymes such as xyloglucan endotransglucosylase/hydrolase (*XTH*; ID101258345) that participates in cell-wall loosening and might benefit cell wall extension, or the plant cell wall protein *SlTFR88* (ID778266) were upregulated by salt stress at the absence of inoculum. By contrast, a pectate lyase (*PL*; ID778293), involved in cell-wall composition, was up-regulated by AXSa06-inoculation in response to salt stress. These changes in transcript levels of genes related to cell-wall modification alterations may be necessary for plant survival under unfavorable conditions, and were probably triggered by ethylene. On the other hand, nitrate reductase (*NR*; ID100736473), encoding the enzyme catalyzing the NAD(P)H-mediated conversion of nitrate to nitrite, which is the rate-limiting step of nitrogen assimilation pathway in higher plants, was significantly down-regulated only in non-inoculated plants due to salt stress. This finding may indicate a transition of metabolism from developmental processes to defense mechanisms. Thus, the inhibition of nitrogen assimilation pathway driven via NR activity, and occurred at high salinity conditions, may be prevented by AXSa06-inoculation.

#### Antioxidant-Related Genes

**Figure 6:**
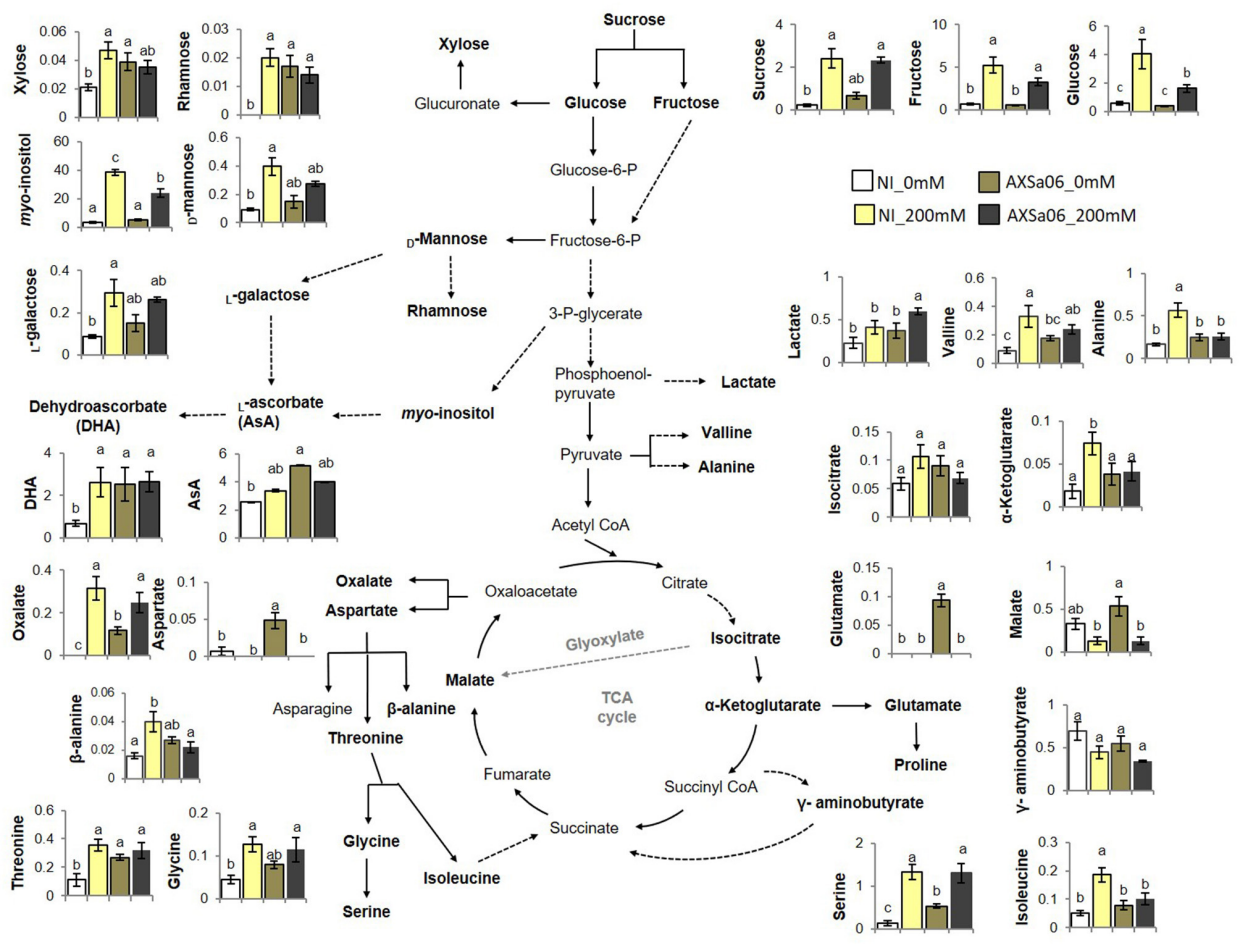
Selected metabolomic profiles of leaves from tomato seedlings inoculated or not with AXSa06 (*Pseudomonas oryzihabitans*) at exposure to 200 mM NaCl. Amounts were expressed as relative abundance based on the relative response compared to internal standard adonitol and are provided in Supplementary Table 12. Ascorbic acid (AsA) and its oxidized form (dehydroascorbate, DHA) were determined spectrophotometrically based on the AO enzyme method. Data are means of five biological replicates ± standard error. Different letters indicate statistically significant differences between treatments based on Duncan's multiple range test (*P* < 0.05). NI, non-inoculated.

Several stress-responsive transcripts were detected among DEGs between control and salt stress treatments. At the absence of inoculum, transcripts related to ROS detoxification and homeostasis including ferredoxin (ID101248335), *AO* (ID101252344), _L_-ascorbate peroxidase (*APX*; ID101258987), and lignin-forming anionic peroxidase (*LPOX*; ID101261260), showed higher expression in stressed plants compared to control ones (Figure 5 and Supplementary Tables 6, 10). By contrast, others such as *myo*-inositol oxygenase (*MIOX*; ID100316875 and ID101254427) and peroxidase 51 (*POX51*; ID101244162) or peroxidase 3 (*POX3*; ID101244376) exhibited lower expression. Similarly, the transcript levels of several stress-induced genes were up-regulated in AXSa06-inoculated plants at exposure to 200 mM NaCl. These included lignin-forming anionic peroxidase (*LPOX*; ID101261260), suberization-associated anionic peroxidase 2 (ID101245316), ferredoxin (ID101266472), *AO* (ID101252861), and inositol oxygenase 1 (ID101263222) (Figure 5 and Supplementary Tables 7, 10, 11). Heat-shock proteins (HSPs) the molecular chaperones that inhibit the stress-mediated denaturation of proteins, were also up-regulated in this study. In particular, five HSPs (ID544024, ID544205, ID101254946, ID101264936, and ID101255223) were induced after salt stress only in non-inoculated plants (Supplementary Table 6). An up-regulation of key ascorbate (AsA) biosynthetic genes including GDP-_L_-galactose phosphorylase (*GGP*, ID101255942), _L_-galactose dehydrogenase (*GalDH*; ID101254135), and _L_-galactono-1,4-lactone dehydrogenase (*GLDH*; ID544206), as well as monodehydroascorbate reductase (*MDHAR*; ID101264360) from the AsA recycling pathway, was also evident in AXSa06-inoculated plants at control conditions compared to non-inoculated ones, supporting an enhancement of AsA-mediated antioxidant defense mechanism in these plants (Figure 5 and Supplementary Table 10). This is also in agreement with an enhanced AsA pool due to inoculation as well be discussed below (Figure 6).

Since phenylpropanoids contribute to plant abiotic stress responses, genes encoding for key enzymes of their biosynthetic pathways were searched in a query to the KEGG database (Supplementary Tables 9, 11). Transcripts coding for 24 genes were found to be up-regulated in response to NaCl treatments in both non-inoculated and inoculated plants (Supplementary Figure 7). The identified genes have diverse functions related to phenylpropanoid-related pathways, being involved in key steps such as _L_-phenylalanine conversion in cinnamic acid [phenylalanine ammonia lyase (PAL2)], or catalysis of the final branches for lignin production (i.e., lignin-forming anionic peroxidase, ID101261260) or suberization (suberization-associated peroxidases, *TMP1, TAP2*, and ID101265511). Interestingly, although several β-glucosidase genes (ID101247513, ID101263519, and ID100191127) were up-regulated due to salt stress in both non-inoculated and inoculated plants, their transcript levels were higher at the absence of NaCl in inoculated compared to non-inoculated plants, suggesting that AXSa06 inoculation has also affected and remodeled phenylpropanoid pathway.

#### Hormone-Related Pathways

The expression levels of hormone-related genes were also differentially regulated by both AXSa06-inoculation and treatment with 200 mM NaCl (Figure 5 and Supplementary Table 10). At the absence of inoculum, salt stress enhanced ethylene biosynthesis as indicated by the significant up-regulation of ACC-synthase 1A (*ACS1A*; ID544028). At the same time S-adenosylmethionine decarboxylase (*SAMDC2*, ID101260400), a key enzyme linking ethylene metabolism and polyamine (spermine and spermidine biosynthesis), previously associated with modified salt responses in tobacco plants (Mellidou et al., 2016, 2020), was also over-expressed under salt stress. The remarkable up-regulation of ethylene biosynthesis through transcription of *ACS1* and *ACO1* due to NaCl treatments was evident at the absence of inoculum (Figure 5). Notably, AXSa06-inoculated plants showed no particular alteration in the expression of ethylene biosynthetic genes upon exposure to salt stress. This is in line with results on ACC accumulation in the leaves (Figure 3C), supporting the impact of the ACC deaminase strain, AXSa06, in inhibiting ethylene synthesis either due to limited precursor availability or ethylene signaling.

Three ABA receptors PYL4 (ID101250944, ID101258963, and ID101256856), required for ABA-mediated responses such as stomatal closure, were significantly down-regulated in non-inoculated plants in response to salt stress (Supplementary Table 6), whilst only one ABA receptor PYL4 (ID101258963) was repressed in inoculated plants (Supplementary Table 7). On the other hand, although the expression levels of some ABA-signaling genes such as protein phosphatase 2C (*PP2C*; ID101249794, ID101258071) that act as negative regulators of ABA-mediated responses, or ABA 8′-hydroxylase (*CYP707A1*; ID101254720), the key enzyme in ABA oxidation/catabolism, were induced upon salt stress regardless the presence of the inoculum (Figure 5 and Supplementary Table 10), but the induction was more drastic in AXSa06-inoculated plants. In particular, these three genes were detected among the top DEGs in AXSa06-inoculated plants in response to 200 mM NaCl (Supplementary Table 7), suggesting a more stunning suppression of ABA signaling due to the PGP strain. Notably however, considering expression levels of these negative regulators of ABA signaling, inoculated plants showed lower expression regardless stress treatment compared to non-inoculated plants. Genes related to other hormone catabolism, such as cytokinin oxidation (cytokinin dehydrogenase; *CKX8*; ID101055579) or gibberellin oxidation (gibberellin 2-oxidase; *GA2ox3*; ID100134888) were up-regulated only in stressed non-inoculated plants, suggesting that these plants probably perceive more stress signals than inoculated ones (Figure 5). Another gene involved in auxin metabolism (indole-3-acetic acid-amido synthetase; *GH3.10*; ID101258277) was significantly down-regulated due to NaCl treatment regardless inoculation with AXSa06.

#### Transcription Factors

Several transcription factor (TFs) families such as WRKY, MYB, and bHLH exert pivotal roles in plant responses to abiotic stress factors. In non-inoculated plants, a broad number of TFs were induced by salt stress, including dehydration-responsive element-binding protein 1A (*DREB1A*; ID109119806 and ID101248897), ethylene-responsive *ERF027* (ID101246746), *WRKY* (ID101264826, ID101246812, ID101261141), *EARLY-RESPONIVE TO DEHYDRATION* protein (*ERD7*; ID101263355), *bHLH92* (ID101245202), and heat stress TF *HsfA2* (ID101255223) (Figure 5 and Supplementary Table 10). A few TFs were also down-regulated in non-inoculated plants treated with NaCl. These included *ERF061* (ID10125880) and *MYB20* (ID101264349). By contrast, only one abiotic stress-responsive TF, *ERF3*-like (ID606713) was up-regulated in inoculated plants under salt stress conditions. Since TFs act downstream the hormonal signals, this finding further supports the previous hypothesis that inoculated plants may receive less stress signals than non-inoculated ones due to the suppression of ABA- and ethylene-activated responses.

#### Other Salt-Responsive Genes

The broad number of secondary structures enables LEA proteins to play multiple roles in abiotic stress, constituting an essential footprint of plant tolerance against irreversible damage (Artur et al., 2019). Homologs of this large protein family were found to be over-expressed at 200 mM NaCl in both non-inoculated (ID544157; Supplementary Table 6) and AXSa06-inoculated plants (ID100750252; Supplementary Table 7). Laccases are also widely present in plants, and have been related to disease resistance and lignin biosynthesis, while their involvement in abiotic stress tolerance is less clear (Liu et al., 2017). In this study, two different laccases (ID101251693 and ID101253711) were positively regulated by salt stress, regardless the presence of inoculum (Supplementary Tables 6, 7). By contrast, galactinol synthase (ID101261450), the key enzyme in the biosynthesis of raffinose, was only up-regulated in AXSa06-inoculated plants exposed to 200 mM NaCl (Supplementary Table 7).

An interesting observation of this study is that the SOS1 protein (ID778208), a putative plasmalemma Na^+^/H^+^ antiporter essential in maintaining ion homeostasis under salinity, through partitioning Na^+^ between plant organs (Olías et al., 2009), was significantly higher (1.4-fold) in AXSa06-inoculated plants compared to non-inoculated plants at control conditions (0 mM NaCl) (Figure 5 and Supplementary Table 10). At salt stress, transcript levels of *SOS1* were induced in non-inoculated plants, while remained at very high levels in inoculated ones. Similarly, the Na^+^ transporter *HKT1*, as well as the Na^+^/H^+^ antiporters (*NHX1* and *NHX2*), were up-regulated due to AXSa06 inoculation at control conditions. These findings indicated a putative improved mechanism of preventing the accumulation of excess Na^+^ in photosynthetic tissues, thus supporting a role of AXSa06 as a priming agent that facilitated plants to tolerate abiotic stress factors.

### Metabolome Reprogramming Under Salt Treatment

In an attempt to generate a comprehensive picture of metabolite reprogramming occurring in AXSa06-inoculated plants at exposure to 200 mM NaCl, we evaluated the abundance of 46 metabolites using GC-MS (Figure 6 and Supplementary Table 12). In total, 25 metabolites showed differential accumulation at the absence of inoculum in plants exposed to salt stress compared to control, whilst at the presence of inoculum, only 14 metabolites differed significantly between stressed and un-stressed plants. Among them, 24 and seven were increased, while one and seven were decreased, in non-inoculated or inoculated plants, respectively.

Of the metabolites with differential accumulation during salt stress, most of them belonged to sugars or amino acids, indicating that their metabolic pathways were predominantly affected by NaCl treatments. In particular, accumulation of sucrose, fructose, glucose, *myo*-inositol, and xylitol, were significantly increased due to salt treatment in both non-inoculated and inoculated plants, but to a greater extent at the absence of inoculum (Figure 6). By contrast, xylose, rhamnose, _D_-mannose, _L_-galactose, allose, sedoheptulose, erythrose, and 2-deoxy-_D_-erythro-pentitol, showed higher accumulation due to salt stress only in non-inoculated plants. As for organic acids identified in this study, oxalate and 2-ketoglutarate increased in non-inoculated plants exposed to stress, whilst lactate and oxalate increased in AXSa06-inoculated ones. However, the accumulation of two other organic acids, malate, and galactarate decreased in inoculated plants. A broad number of amino acids increased as a response to salt stress at the absence of inoculum, including alanine, valine, isoleucine, glycine, serine, threonine, and cystathionine. Among them, only serine was induced in AXSa06 plants, while two other amino acids, aspartate and glutamate decreased at exposure to 200 mM NaCl. No particular changes in AsA contents due to NaCl application were recorded, but inoculated plants had significantly higher AsA levels at control conditions (Figure 6). Furthermore, similar to the majority of sugars, DHA, the oxidized form of AsA representing an indicator of cellular redox homeostasis was enhanced at NaCl treatment of non-inoculated plants, but remained unaltered when the inoculum was applied.

Collectively, these metabolomic findings suggest that salt stress imposed far less metabolomic reprogramming when plants were inoculated with AXSa06. The metabolomic profile of non-inoculated compared to inoculated plants at control conditions (0 mM NaCl) revealed a vast number of metabolites over-accumulated in AXSa06 treatments (Figure 6 and Supplementary Table 12). In particular, the sugars 2-desoxy-inosose, 2-deoxy ribose and the cell-wall related xylose and rhamnose, the sugar alcohols erythritol and 2-deoxy ribitol, the amino acids aspartate, threonine, serine and glutamate, as well as the acids 2-deoxy ribonate, oxalate and AsA, were all increased owing to AXSa06 inoculation, indicating a probably reprogramming of metabolism that may support plant adaptation against salt. This pattern was further supported by results concerning sucrose, mannose, valine, glycine, and malate, although differences were not statistically significant.

## Discussion

Plants have evolved symbiotic interactions with soil microbes that can alter plant phenotypic plasticity in a broad range of traits during plant growth or in response to changing environment (Goh et al., 2013). PGPR can enhance plant growth via direct or indirect mechanisms, including nutrient mobilization, solubilization and bio-availability, phyto-hormone biosynthesis, and induced systemic resistance (Yang et al., 2009; Pérez-Jaramillo et al., 2018; Gamez et al., 2019). Along with their plant-growth-promoting properties, and their ability to protect from phytopathogenic infections, some PGPR are also capable of enhancing tolerance to abiotic stresses such as salinity and drought (Yang et al., 2009). The exact mode of action by which PGPR affect plant growth under adverse environmental conditions may be both plant species- and strain-specific, as highlighted by the vast number of studies in different plants (Liu et al., 2017; Chauhan et al., 2019; Gamez et al., 2019; Safdarian et al., 2019). The present study demonstrated that AXSa06, a *Pseudomonas oryzihabitans* strain previously isolated from extreme saline ecosystem, able to consume ACC, to produce IAA and siderophores, as well as to solubilize inorganic P (Leontidou et al., 2020), efficiently colonized the rhizosphere of tomato seedlings and promoted plant growth under salt stress. Inoculations with the strain seem to impose plants to a primed state, at which they are able to respond more robustly to abiotic stress owing to the earlier increased and more efficient activation of the defense response, as previously reported (Conrath, 2011). The investigation of key genes and metabolic pathways further supported the primed state of AXSa06-inoculated plants prior to NaCl treatments that probably has led to a less disturbed metabolism after exposure to stress, by dampening stress signals, contributing to AXSa06-mediated tolerance. This is further supported by the GO enrichment analysis demonstrating that *Pseudomonas oryzihabitans* AXSa06 strain was able to down-regulate transcripts related to peptidase regulator or inhibitor activity that were otherwise found to be stimulated by salt treatment in non-inoculated plants due to protein degradation and synthesis of defensive proteins.

### AXSa06 Stimulated Antioxidant Mechanism

As a primary stress response to NaCl treatments, a burst in ROS production is expected, resulting in extensive cellular damage if not tightly regulated by the antioxidant scavenging systems in plants (Gémes et al., 2016; Noctor et al., 2016). Several antioxidant enzymes, including CAT, SOD, APX, and POX are known to increase in response to salt stress in order to mitigate salt-induced damage as has been reported in many plant species, including tobacco (Mellidou et al., 2016) and cotton (Hamani et al., 2020). Previous studies also reported an up-regulation of ROS scavenging activities and of antioxidant components accumulation due to bacteria inoculation under salt conditions (Bharti et al., 2016; Chen et al., 2016). Nevertheless, in this study, the prior-to-stress stimulation of antioxidant machinery (enzymatic and non-enzymatic) in AXSA06 plants may account for the improved salt adaptation, without any additional energy cost to support the protection of cellular redox homeostasis when stress is applied. In other words, strain inoculation may have imitated the effect of mild oxidative stress, similar to what is expected during salt stress. This primed state of AXSa06-inoculated plants is supported by the increased content of AsA and its oxidized form, DHA, as well as the enhanced activities of antioxidant enzymes, including POX and CAT that scavenge H_2_O_2_, compared to non-inoculated ones at control conditions. Within this context, the induction of antioxidant defense mechanism probably has occurred to limit down lipid peroxidation due to inoculation in non-stressed plants, which, however, did not show any visual symptom of oxidative stress. Although MDA content has been mostly considered as an oxidative stress marker triggering a wide range of other developmental signals, it can be also beneficial for plants in a number of ways participating in the induction of antioxidant defense or membrane repair (Tounekti et al., 2011; Kurutas, 2016; Morales and Munné-Bosch, 2019). Since photosynthetic process was not inhibited, plant growth was vigor, and ROS were probably efficiently removed in the inoculated plants, these plants may experience a mild oxidative boost just enough to increase their alertness, for amplified defense responses upon the onset of stress treatment. In other words, lipid peroxidation in inoculated plants may be beneficial—to a certain extent—to stimulate their alertness prior to stress. Tounekti et al. (2011) also reported that when MDA accumulation is elevated, rosemary plants can withstand salt-induced oxidative stress, by activating the xanthophyll cycle-dependent dissipation of excess excitation energy in leaves.

Due to their non-specific reactions with different ROS, POX enzymes participate in a broad number of physiological functions, including auxin catabolism, suberization, lignifications, cell wall elongation, and pathogen attack (Malviya et al., 2020). Previously, homologs of different *POX* genes have been implicated in lignin and xylan accumulation in *Arabidopsis* through ROS signaling (Cosio et al., 2017), as well as in salt tolerance in soybean (Jin et al., 2019). Herein, a lignin-related *POX* was up-regulated at exposure to 200 mM NaCl regardless inoculation, but a suberization-associated *POX* only at the presence of the PGPR strain. Although suberization primary occurs in the roots, this adaptive anatomical process is important for the prevention of apoplastic flow of toxic Na^+^ under salt stress (Shabala et al., 2012).

In response to abiotic stress, plants synthesize a broad number of secondary metabolites possessing or inducing antioxidant properties to alleviate the over-accumulation of ROS and inhibit cell membrane peroxidation (Baba et al., 2017; Sharma et al., 2019). In particular, upon stress treatments, the phenylpropanoid pathways are commonly activated through the up-regulation of the transcript levels of genes encoding key biosynthetic enzymes like *PAL*, chalcone synthase and isomerase (Perin et al., 2019). In line with previous reports, several phenylpropanoid-related genes were up-regulated upon salt treatment, but this occurred regardless the presence of AXSa06. Although phenolic compounds were not determined in this study, the slightly increased transcript levels of the related genes at control conditions due to AXSa06 compared to non-inoculated plants, suggested that plants employed these metabolites as a communication tool to attract desirable symbiotic rhizobacteria, as have already been demonstrated between legumes and nitrogen fixing bacteria (Biała and Jasiński, 2018). On the other hand, β-glucosidases have an interesting role in abiotic stresses, particularly dehydration through ABA, by hydrolyzing secondary metabolites that are stored in inactive glycosylated forms (Baba et al., 2017). These enzymes have crucial functions in plants in response to altered environmental cues, including the glycosylation of secondary metabolites that are stored in inactive glycosylated forms and the activation of lignin precursors (Rouyi et al., 2014). The higher transcript levels of various β-glucosidase genes due to AXSa06 may contribute to the enhanced stress tolerance of inoculated plants, by releasing glucose which could serve as an alternative source of energy during insufficient photosynthesis.

AsA has numerous functions linked to oxidative relief under stress conditions, including the cellular scavenging of H_2_O_2_ via APX, or the apoplastic regulation of the AsA redox state via AO, which is important for stress perception and orchestration of defense responses (Mellidou and Kanellis, 2017). Therefore, AsA pool size and homeostasis may act as the signal of the oxidative injury occurring under stress, and play a pivotal role in controlling plant responses to environmental changes (Pignocchi et al., 2003). Transcript levels of *APX* were up-regulated at the absence of inoculum as a response to salinity, contributing to the significant increase in DHA accumulation in these plants, but no similar pattern was observed in AXSa06 treatments, indicated that these plants may sense less oxidative stress. If these oxidized forms cannot be regenerated by monodehydroascorbate reductase (MDHAR) and dehydroascorbate reductases (DHAR) in the AsA-GSH cycle, then AsA is permanently lost (Noctor et al., 2016). The increased transcript levels of AsA biosynthetic and recycling genes, combined with the accumulation of AsA itself and its precursor, _L_-galactose at control conditions, indicated that inoculations with AXSa06 enhanced the AsA-mediated capacity of plants to scavenge ROS, further supporting the role of this strain as a priming agent against stress. This is in line with previous studies demonstrating that the regulation of antioxidant enzyme activities by PGPR exert a pivotal role in stimulating strain-mediated salt tolerance of plants (Safdarian et al., 2019).

### AXSa06 Altered Amino Acid and Sugar Metabolism Imitating Salt Stress Responses

Although the phenomenon has been known for decades, the molecular basis of PGPR-mediated priming against abiotic stresses is far from being clearly elucidated. The over-accumulation of osmoprotectants such as proline is considered as the most usual mechanism of osmotic stress avoidance. Indeed, at exposure to salt stress, proline levels highly increased in both inoculated and non-inoculated plants, and similar to results from others (Kim et al., 2014; Liu et al., 2017), transcript levels of *P5CS1*, a key gene from proline biosynthetic pathway leading to osmoregulation, was also stimulated, independently of inoculation. These findings indicated that AXSa06-mediated stress tolerance is probably not related to a more efficient osmoregulatory activity in inoculated plants, at least due to proline.

Carbohydrates, including hexoses (fructose and glucose), disaccharides (sucrose and trehalose), and oligosaccharides (raffinose), are well-known to control ionic balance, maintaining cell turgor, while some of them are also considered as signaling molecules or even as ROS detoxification molecules in response to salt stress (Keunen et al., 2013; Patel et al., 2020). In this study, inoculated plants showed enhanced accumulation of certain sugars prior to stress application (erythrose, xylose, 2-deoxy -ribose, and 2-desoxy-inosose), or displayed no particular induction of some other sugars (sucrose and galactose) in exposure to salt stress similar to those observed in non-inoculated plants. These results support the notion that the metabolism of AXSa06-inoculated plants was less disturbed due to salt. The synthesis of other compatible solutes, such as raffinose family oligosaccharides (RFOs), represents an additional mechanism used by plants to adapt during the adverse effects of stressful conditions (Liu et al., 2021). Their mode of action involves the protection of membranes from hydroxyl radicals, as they are found between the head groups of lipids. The formation of galactinol from *myo*-inositol is catalyzed by galactinol synthase, representing the first enzyme that commits carbon toward RFO formation (Vinson et al., 2020). Transcript levels of this gene were only significantly induced in AXSa06-inoculated plants exposed to 200 mM NaCl, indicating an additional role of galactinols as osmoprotectants and stabilizers of cellular membranes, but also as scavengers of ROS under excess salt (Nishizawa et al., 2008). Although none of these compounds were quantified in this study, the decreased levels of *myo*-inositol—the substrate of galactinol synthase—upon AXSa06 inoculation due to stress, may reflect the better adaptation of inoculated plants through this pathway.

Notably, the level of four amino acids (aspartate, threonine, serine, and glutamate) all increased owing to AXSa06 inoculation under control conditions indicating prominent changes in amino acids metabolism. Previously, the accumulation of free amino acids in combination with the higher transcript levels of related biosynthesis genes have been linked with salt tolerance, suggesting a crucial role of amino acid metabolism in stress tolerance (Zhang et al., 2017; Liu et al., 2020). Among these amino acids, the higher abundance of aspartate and glutamate mediated by AXSa06 inoculation at the absence of stress is of particular interest. Both molecules are involved in asparagine biosynthesis that is a major part of amino acid and nitrogen metabolism in plants (Curtis et al., 2018), strongly regulated by salt stress (Rashmi et al., 2019), and may operate as a pool of precursors for this metabolic branch, when plants exposed to NaCl. Asparagine has been also implicated in the early responses against salt stress in sugar beet (Liu et al., 2020), and can be further used as precursor in methionine and glutathione biosynthesis, that are able to scavenge ROS (Noctor et al., 2012). On the other hand, glutamate can be used to produce proline and gamma-aminobutyric acid (GABA) (Liu et al., 2020). In *Arabidopsis*, knockout mutants with T-DNA insertion in asparagine synthetase 2 gene exhibited limited salt tolerance and impaired nitrogen assimilation and translocation by the NaCl treatment relative to the wild-type (Maaroufi-Dguimi et al., 2011). In spite of the fact that GABA has been reported to alleviate salt stress injury in tomato seedlings by modifications in ion flux, amino acid synthesis and key enzyme expression (Wu et al., 2020), no similar relation was found in this study. Moreover, the increased levels of threonine and serine after AXSa06 inoculation revealed the modification of “glycine, serine, and threonine metabolism” pathway in response to inoculum at control conditions. Similarly, in non-inoculated plants exposed to salt stress, genes related to this pathway, such as *TD* and *TSB* were significantly up-regulated, with a concomitant increase in metabolic fluxes, whilst *ASRGL2* involved in asparagine metabolic pathway was down-regulated in non-inoculated plants, probably to support fluxes toward threonine and serine that were enhanced. These findings further support the notion that at control conditions AXSa06 exerted a similar response of primary metabolism related to amino acids as non-inoculated plants at the presence of NaCl, suggesting a kind of mild stress due to the inoculum that primed plants prior stress exposure.

No particular changes in tricarboxylic acid cycle were observed between non-inoculated and AXSa06-inoculated plants at metabolic or transcriptomic level in response to salt stress. This could be probably explained by an earlier induction of this pathway during the first days of stress application, as previously reported in sugar beet (Liu et al., 2020), that we were not able to detect at long-term (7 days) salt stress. An early down-regulation of pathways related to energy metabolism could serve as the signal for the transition from plant growth to the enhancement of defense mechanisms to cope with the stress factor (Bandehagh and Taylor, 2020). On the basis of these considerations, future studies should focus on discriminating early and late responses to salt stress, as well as providing clues on their importance in alleviating stress injury.

### AXSa06 Regulates Hormone Production and Signaling

Ethylene and ABA are considered as key phytohormones, acting in concert as double-edged swords to coordinate stress responses to various abiotic factors (Müller, 2021). Several lines of evidence over the last decades suggest that salt stress stimulates ethylene production in plants, ultimately inhibiting plant growth and development (Riyazuddin et al., 2020). In this regard, PGPR that possess ACC deaminase activity are considered to increase salt tolerance of plants through inhibiting ethylene production by consuming ACC, and thus enhancing plant growth (Liu et al., 2017; Safdarian et al., 2019), although this is not their sole mode of action accounting for salt tolerance. In this study, root ACC content decreased in inoculated plants compared to non-inoculated ones exposed to salt stress, highlighting the positive effect of inoculation with the ACC deaminase-containing strain on inhibiting the ACC flux toward ethylene biosynthesis. Nonetheless, its effect in the above-the-ground parts is less clear. Under salt stress, the reduction in ACC content at the absence of inoculum may be attributed to its consumption to produce ethylene due to NaCl treatments, whilst no particular changes were observed at the presence of inoculum, probably because the ethylene signal from the roots is inhibited due to the consumption of ACC from the strain. During salt stress, *ACS3* and *ACO4* displayed no particular up-regulation, while *ACS1A* and *ACO1* were upregulated, but to a lesser extent when plants were inoculated with ASXa06. This data suggests that ethylene production, and in turn, ethylene signaling, are probably inhibited by the AXSa06 inoculum. Although the ethylene production has not been measured *in vivo*, ethylene determination in the excised leaves of AXSa06-inoculated plants further confirmed that there was less ethylene signaling-sensitivity during salt stress, and consequently less feedback of ethylene on its own biosynthesis, as highlighted by the lack of major modifications in the expression of ethylene-biosynthetic genes. The reduced ethylene production could also account for the observed higher ACC accumulation under salt stress compared to non-inoculated plants, suggesting that ACC was not consumed for ethylene production in inoculated plants. All these findings need to be further confirmed to elucidate the exact role of ethylene in PGPR-mediating tolerance. Previously, it has been demonstrated that ethylene signaling and homeostasis is linked with certain PGPR-mediated responses in modulating plant stress tolerance (Chen et al., 2013; Poupin et al., 2016; Liu et al., 2017). The dramatic increase in root MACC content of inoculated plants exposed to 200 mM NaCl indicated that the major part of ACC was used for MACC formation rather than ethylene biosynthesis, thus evidencing that the ethylene accumulation in the roots is inhibited resulting to lower levels of ethylene signaling in the above-the ground-parts. MACC is an end product, providing a potential mechanism to control ethylene biosynthesis (Van de Poel et al., 2014), while its exact mode of action under salt stress requires further investigation.

ABA and ethylene have antagonistic relations in the control of stomatal opening and closure (Müller, 2021). In this study, the expression of some ABA signaling components such as protein phosphatases 2C that act as negative regulators of ABA-mediated responses, or ABA 8′-hydroxylase, the key enzyme in ABA oxidation/catabolism, were more drastically induced under salt stress conditions owing to AXSa06 inoculation compared to non-inoculated plants, suggesting a more vivid suppression of ABA signaling and accumulation. The up-regulation of these genes has been previously correlated to enhanced salt tolerance, as ABA regulates the integration of stress signals, especially the ethylene-mediated ones (Campobenedetto et al., 2020). At the same time, the lower absolute expression of these ABA-related genes at both control and stress conditions in the presence of AXSa06 may be related to the lower accumulation of ABA, that could have otherwise led to stomata closure, disturbing photosynthesis (Jakab et al., 2005; Vishwakarma et al., 2017). Previously, the ABA-signaling components have been found to regulate both fast and slow ABA-mediated responses to tackle dehydration under salinity (Vishwakarma et al., 2017). Therefore, the beneficial impact of AXSa06 inoculations in plant salt tolerance could be possibly due to the more rapid and efficient suppression of both ethylene- and ABA-mediated stress signals.

Several TF families, including WRKY, bHLH, and DREB, have been previously demonstrated to orchestrate plant stress reactions against abiotic stress (Golldack et al., 2011; Liu et al., 2017). In the present study, non-inoculated plants showed an induction of these TFs when exposed to salt stress, which may be regarded as a defense reaction to ameliorate stress damage, by modulating osmotic and ROS homeostasis. In contrast to other studies on PGPR stains (Liu et al., 2017; Safdarian et al., 2019), these stress-responsive TFs, similar to ERFs that are located downstream the genes of the ethylene signaling pathway, were not upregulated in AXSa06-inoculated plants exposed to stress compared to non-stress conditions. These observations suggest that AXSa06 prevented the induction of the stress-induced ethylene biosynthesis, and further damped ABA synthesis and signaling during salt stress. In other words, AXSa06 minimizes the activity of two major abiotic stress hormones, and thus inoculated plants displayed less hormone-mediated responses due to salt stress.

As AXSa06 is able to produce IAA *in vitro* (Leontidou et al., 2020), the probable enhanced strain-mediated IAA production may serve as an important signal for plants to cope with salt stress. Although salt stress imposed IAA modifications at the transcript level regardless AXSa06 inoculation, there was no clear link between the PGP strain and IAA homeostasis. In particular, an indole-3-acetic acid-amino synthetase, responsible for the inhibition of free IAA accumulation (Ding et al., 2008), was down-regulated regardless the presence of the strain. Genes of the *GH3* (Gretchen Hagen 3) family are involved in IAA homeostasis through catalysis of auxin conjugation and by binding free IAA to amino acids (Liu et al., 2016). In rice, some *GH3* genes were found to be related to stress responses, i.e., by decreasing the endogenous IAA content enhancing drought tolerance (Zhang et al., 2009), or through modulating ABA levels under drought and freezing stress (Du et al., 2012). One of the key genes in the tryptophan-dependent pathway of auxin biosynthesis is indole-3-pyruvate monooxygenase *YUCCA*, which convert indole-3-pyruvic acid to bioactive IAA (Zhao et al., 2001). Its transcript levels were also found to be down-regulated regardless the presence of the PGP strain, further supporting the disruption of IAA homeostasis predominantly due to salt stress, and not owing to AXSa06. Taken together, it is evident that AXSa06 did not have a direct impact on IAA metabolism, while the intriguing crosstalk between IAA, ethylene and ABA metabolism, as well as other signal networks, needs to be further elucidated using hormone-deficient mutants.

### AXSa06 Alleviates Ion Toxicity

On the other hand, salt stress evokes ion toxicity due to the higher Na^+^ accumulation within plant tissues. Several ion transporters have been shown to regulate Na^+^ influxes into plant cells, by inducing Na^+^ circulation and sequestration (Liu et al., 2017). For instance, overexpression of *NHX* encoding a tonoplast Na^+^/H^+^ antiporter enhanced salt tolerance of *Arabidopsis* plants (Zhang and Blumwald, 2001), while its expression can be induced by inoculations with *Bacillus amyloliquefaciens* in maize (Chen et al., 2016) and *Arabidopsis* (Liu et al., 2017), but not in rice (Nautiyal et al., 2013), indicating that transcriptome responses are species-dependent. Another Na^+^ transporter with high affinity for K^+^, the sodium transporter *HKT1*, is also known to be induced in plants due to inoculations with *Bacillus* species, thereby limiting Na^+^ accumulation and toxicity in plant cells (Chen et al., 2016; Liu et al., 2017). In this study, the lower Na^+^ contents measured in leaves of AXSa06-inoculated plants may be due to the induction of *NHX1, NHX2*, and *HKT1* transcript levels due to bacterial inoculation prior to stress application that enabled an efficient mechanism of Na^+^ detoxification for coping with salt stress in tomato seedlings (Bharti et al., 2016; Chen et al., 2016). Furthermore, the SOS signaling pathway has been proposed to mediate cellular signaling under salt stress, in order to maintain ion homeostasis (Ji et al., 2013). The expression pattern of *SOS1* was induced both at control and NaCl treatments at inoculations with the *Pseudomonas oryzihabitans* strain, further contributing to the observed salt tolerance of inoculated plants. Taking into consideration that the ABA signaling pathway was not enhanced in the presence of bacterium, it is evident that this SOS-mediated pathway acts independent of ABA to help plants withstand Na^+^ toxicity during salt stress through an intricate mechanism that needs to be further explored.

### AXSa06 Mitigates the Negative Effect of Salt Stress on Photosynthesis

The increased net photosynthetic rate and chlorophyll content of the AXSa06-inoculated plants compared to non-inoculated plants under salt stress can be attributed to the ACC-deaminase activity due to the presence of inoculum in the rhizosphere that can mitigate the effect of salt on photosynthesis, as previously reported (Chauhan et al., 2019). In tomato, ethylene levels can have a negative impact on plant photosynthesis (Ceusters and Van de Poel, 2018), which is probably ameliorated by the ASXa06 inoculum. Furthermore, the enhanced net photosynthesis upon salt stress in the presence of the PGP strain may be related to the suppression of ABA signals that would otherwise had led to stomatal closure and photosynthesis suppression (Yu et al., 2020). This is in accordance with the down-regulation of the photosynthetic KEGG pathway, as well as the transcript levels of *NR*, at the absence of inoculum under salt stress. *NR* is the key enzyme for the reduction of $NO_{3}^{-}$ to $NO_{2}^{-}$ in nitrogen assimilation in plants cells, which coordinates with the rate of photosynthesis and the availability of C skeletons. It can also function as a key enzymatic source of nitric oxide, which then regulates plant growth and resistance to abiotic stresses (Fu et al., 2018). Thus, a better assimilation of both N and C may be evident at the presence of AXSa06, as further illustrated by the more active ruBisCO.

## Conclusion

**Figure 7:**
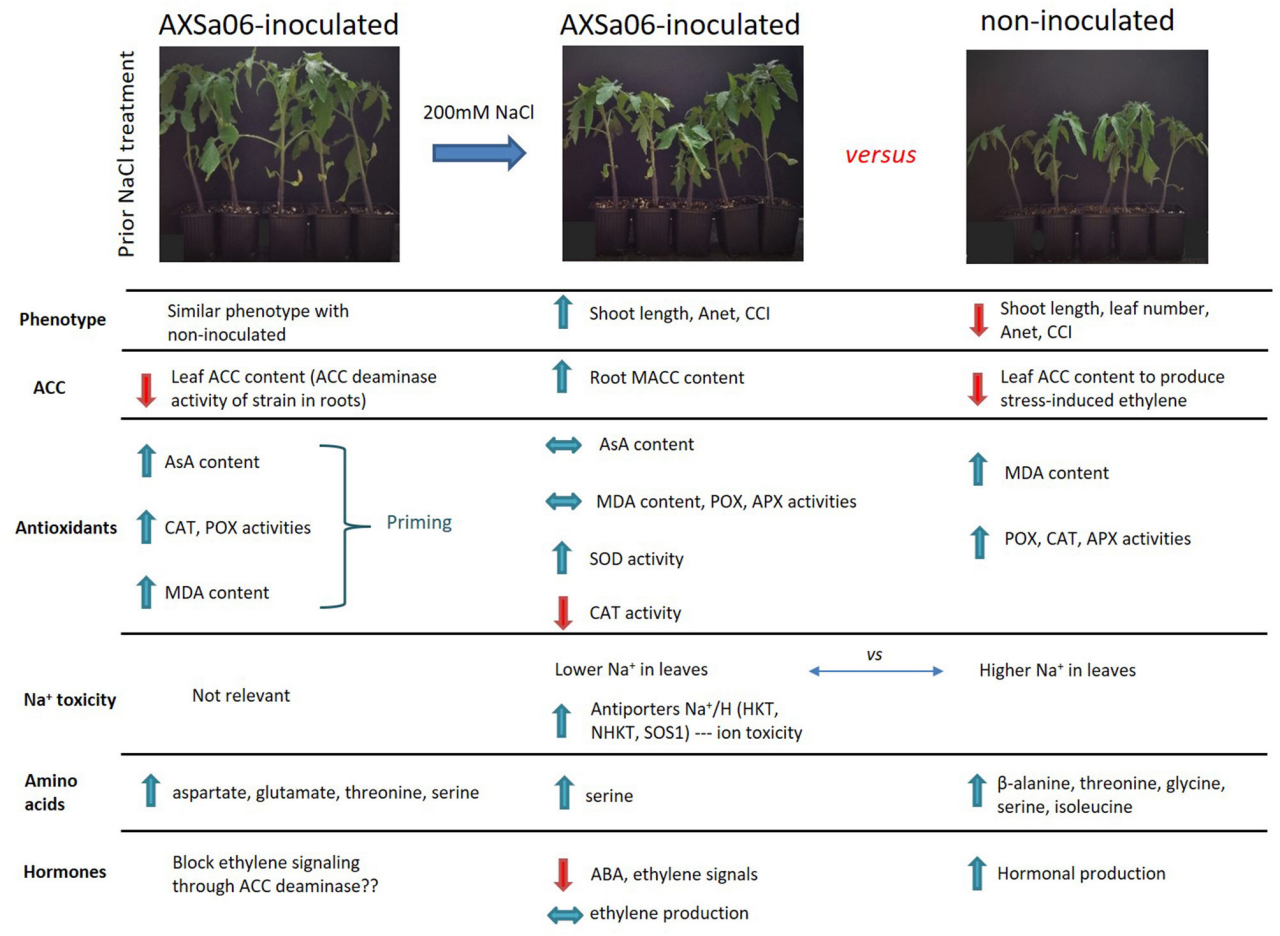
Proposed model for AXSa06-mediated salt tolerance in tomato seedlings exposed to 200 mM NaCl. Inoculation with AXSa06 imposed plants at a pre-conditioned state prior to stress application, by stimulating their antioxidant defense response, by blocking ethylene signals through the activity of ACC deaminase, as well as by enhancing amino acid synthesis. Upon salt stress exposure, AXSa06-inoculated plants showed improved growth and photosynthetic traits, as well as a reduced tendency for Na^+^ uptake which is accompanied by enhanced transcript levels of Na^+^/H^+^ antiporters. Furthermore, AXSa06 represses stress-inducing signals through a dampened ethylene and ABA metabolism, as well as reduced activation of downstream transcription.

Collectively, our results reinforce the hypothesis that inoculations with the *Pseudomonas oryzihabitans* strain (AXSa06) significantly increased the alertness of inoculated plants, imposing them on a pre-conditioned state prior to stress application that render them primed for amplified defense responses. The primed state resulted from an enhancement of antioxidant adaptive mechanism, accompanied by a reduced tendency for Na^+^ uptake (Figure 7). Furthermore, AXSa06 represses stress-inducing signals through a dampened ethylene and ABA metabolism and a reduced activation of downstream transcription factors when stress is applied. The differential modulation of the identified metabolites and transcripts is dependent on the perceived stimuli originated from AXSa06 inoculation that enables plants to fine-tune their defense responses. The identified signatory molecules including AsA, and the amino acids aspartate, threonine, serine, and glutamate, may serve as possible biomarkers for PGPR priming in tomato. Furthermore, inoculations with AXSa06 alleviate the negative impact of salinity on photosynthetic machinery and carbon assimilation, through a more active *ruBisCO* and *NR*, involving an efficient mechanism of Na^+^ detoxification. The molecular mechanism that links microbial signals with plant phenotypic plasticity and fitness merits further investigation to identify possible links between PGPR-priming and epigenetic memory to enhance plant tolerance under diverse environmental cues.

## Data Availability Statement

The original contributions presented in the study are publicly available. This data can be found here: NCBI repository, accession number: PRJEB42497.

## Author Contributions

This study was designed and conceived by IM and KK. Stress experiments, plant growth, physiological, and biochemical evaluation was conducted by IM and AP. Determination of Na^+^ was conducted by AP. Ethylene-metabolite related work was carried out by BVP, while ethylene determination by EK. Transcriptomic analyses were performed by IM, KL, and SG. Metabolomic analyses were undertaken by AA and AP. Data interpretation and manuscript preparation were done by IM. This whole project was supervised by IM and KK. All authors interpreted the results, read, and approved the final manuscript.

## Conflict of Interest

The authors declare that the research was conducted in the absence of any commercial or financial relationships that could be construed as a potential conflict of interest.

## Publisher's Note

All claims expressed in this article are solely those of the authors and do not necessarily represent those of their affiliated organizations, or those of the publisher, the editors and the reviewers. Any product that may be evaluated in this article, or claim that may be made by its manufacturer, is not guaranteed or endorsed by the publisher.
