# Supplementary material for: Comparative Transcriptomics and Metabolomics Reveal an Intricate Priming Mechanism Involved in PGPR-Mediated Salt Tolerance in Tomato
Source: Front Plant Sci. 2021 Aug 17;12:713984. doi: 10.3389/fpls.2021.713984 (PMC8416046; doi:10.3389/fpls.2021.713984)
Supplement: Supplementary file 2 [file Data_Sheet_1.docx]

**Comparative transcriptomics and metabolomics reveal an intricate priming mechanism involved in PGPR-mediated salt tolerance in tomato**

**Ifigeneia Mellidou^1*^, Aggeliki Ainalidou^2^, Anastasia Papadopoulou^2^, Kleopatra Leontidou^2^, Savvas Genitsaris^3^, Evangelos Karagiannis^4^, Bram Van de Poel^5^, Katerina Karamanoli^2^***

**Supplementary Files**

**Supplementary Fig. 1.** Tomato seedlings inoculated or not with AXSa06 (*Pseudomonas oryzihabitans*) at exposure to 200 mM NaCl for seven days.

**Supplementary Fig. 2.** Ethylene production in excised leaves of tomato seedlings inoculated or not with AXSa06 (*Pseudomonas oryzihabitans*) at exposure to 200 mM NaCl for seven days. Data are means of five biological replicates ± standard error. Different letters indicate statistically significant differences between treatments based on Duncan’s multiple

**Supplementary Fig. 3.** GO annotations of differentially expressed genes upon exposure to 200 mM NaCl. (A) up-regulated DEGs in non-inoculated plants, (B) up-regulated DEGs in AXSa06-inoculated plants, (C) down-regulated DEGs in non-inoculated plants, and (D) down-regulated DEGs in AXSa06-inoculated plants.

**Supplementary Fig. 4.** KEGG pathway enrichment of differentially expressed genes upon exposure to 200 mM NaCl. (A) up-regulated DEGs in non-inoculated plants, (B) up-regulated DEGs in AXSa06-inoculated plants, (C) down-regulated DEGs in non-inoculated plants, and (D) down-regulated DEGs in AXSa06-inoculated plants.

**Supplementary Fig. 5.** Cluster analysis on differential expression in non-inoculated plants exposed to 200 mM NaCl. The color key represents normalized log2 FPKM values. The top dendrogram shows the relationships among treatments (salt stress) and the side dendrogram relationships among genes.

**Supplementary Fig. 6.** Cluster analysis on differential expression in AXSa06-inoculated plants exposed to 200 mM NaCl. The color key represents normalized log2 FPKM values. The top dendrogram shows the relationships among treatments (salt stress) and the side dendrogram relationships among genes.

**Supplementary Fig. 7.** Heatmap representing key genes from the phenylpropanoid pathway differentially expressed between non-inoculated versus AXSa06-inoculated plants in response to 200 mM NaCl. The color key represents normalized log2 FPKM values.

**Supplementary Table 1**. Summary of transcriptome sequencing data and mapping ratio to the tomato reference genome.

**Supplementary Table 2**. Expressed genes with FPKM>1 found in non-inoculated plants at 0 mM NaCl.

**Supplementary Table 3.** Expressed genes with FPKM>1 found in non-inoculated plants at 200 mM NaCl.

**Supplementary Table 4**. Expressed genes with FPKM>1 found in AXSa06-inoculated plants at 0 mM NaCl.

**Supplementary Table 5**. Expressed genes with FPKM>1 found in AXSa06-inoculated plants at 200 mM NaCl.

**Supplementary Table 6**. List of DEGS with │log2(fold change) > 1.5│, and corrected p values ≤ 0.01, detected in non-inoculated plants at 0 mM *versus* 200 mM NaCl.

**Supplementary Table 7**. List of DEGS with │log2(fold change) > 1.5│, and corrected p values ≤ 0.01, detected in AXSa06-inoculated plants at 0 mM *versus* 200 mM NaCl.

**Supplementary Table 8**. GO annotations of DEGs in non-inoculated and AXSa06-inoculated plants upon exposure to 200 mM NaCl.

**Supplementary Table 9**. KEGG enrichment analysis of DEGs in non-inoculated and AXSa06-inoculated plants upon exposure to 200 mM NaCl.

**Supplementary Table 10**. FPKM values of key genes of interest related to photosynthesis, amino acid and antioxidant metabolism, plant growth, hormone metabolism, ion transporters and transcription factors, in the leaves of tomato seedlings inoculated with AXSa06 at exposure to 200 mM NaCl, based on log2 transformed FPKM values.

**Supplementary Table 11**. FPKM values of key genes of interest related to phenylpropanoid pathway in the leaves of tomato seedlings inoculated with AXSa06 at exposure to 200 mM NaCl, based on log2 transformed FPKM values.

**Supplementary Table 12**. Metabolomic profiles of leaves from tomato seedlings inoculated or not with AXSa06 at exposure to 200 mM NaCl. Amounts were expressed as relative abundance based on the relative response compared to internal standard adonitol. Data are means of five biological replicates ± standard error. Different letters indicate statistically significant differences between treatments based on Duncan’s multiple range test (P < 0.05).
