# Supplementary figures and images for: Comparative Transcriptomics and Metabolomics Reveal an Intricate Priming Mechanism Involved in PGPR-Mediated Salt Tolerance in Tomato
Source: Front Plant Sci. 2021 Aug 17;12:713984. doi: 10.3389/fpls.2021.713984 (PMC8416046; doi:10.3389/fpls.2021.713984)

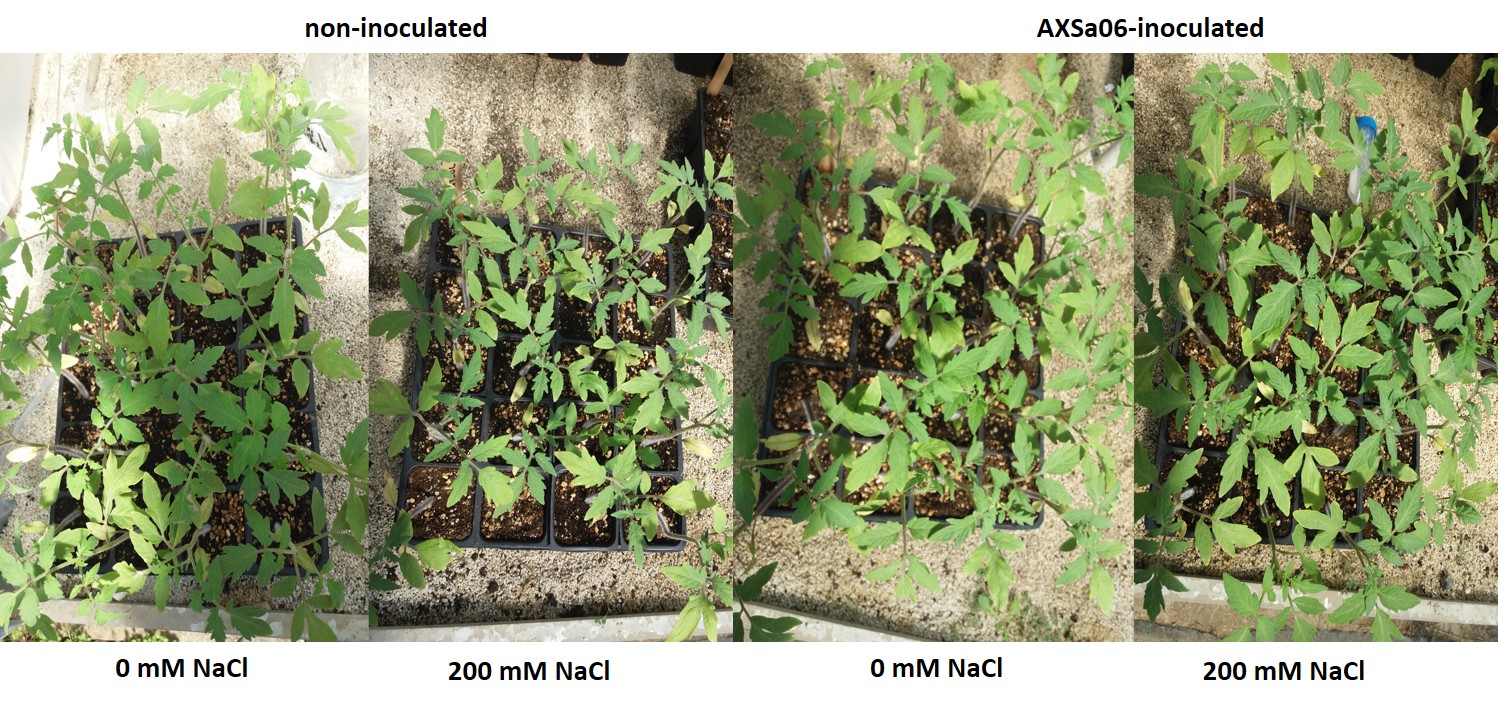

Supplement: Supplementary file 3 [file Image_1.JPEG]

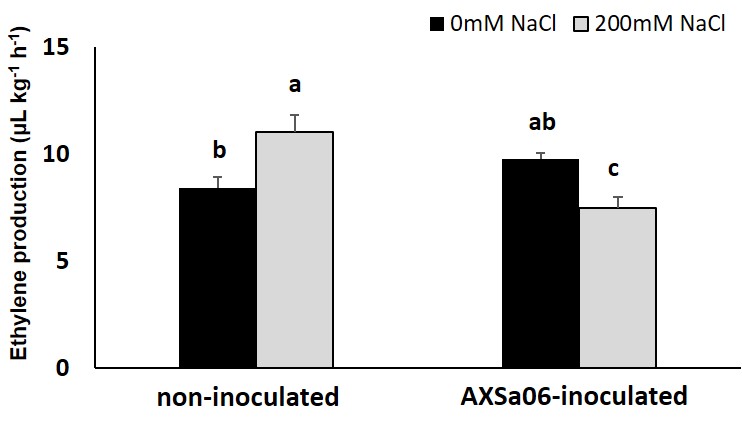

Supplement: Supplementary file 4 [file Image_2.JPEG]

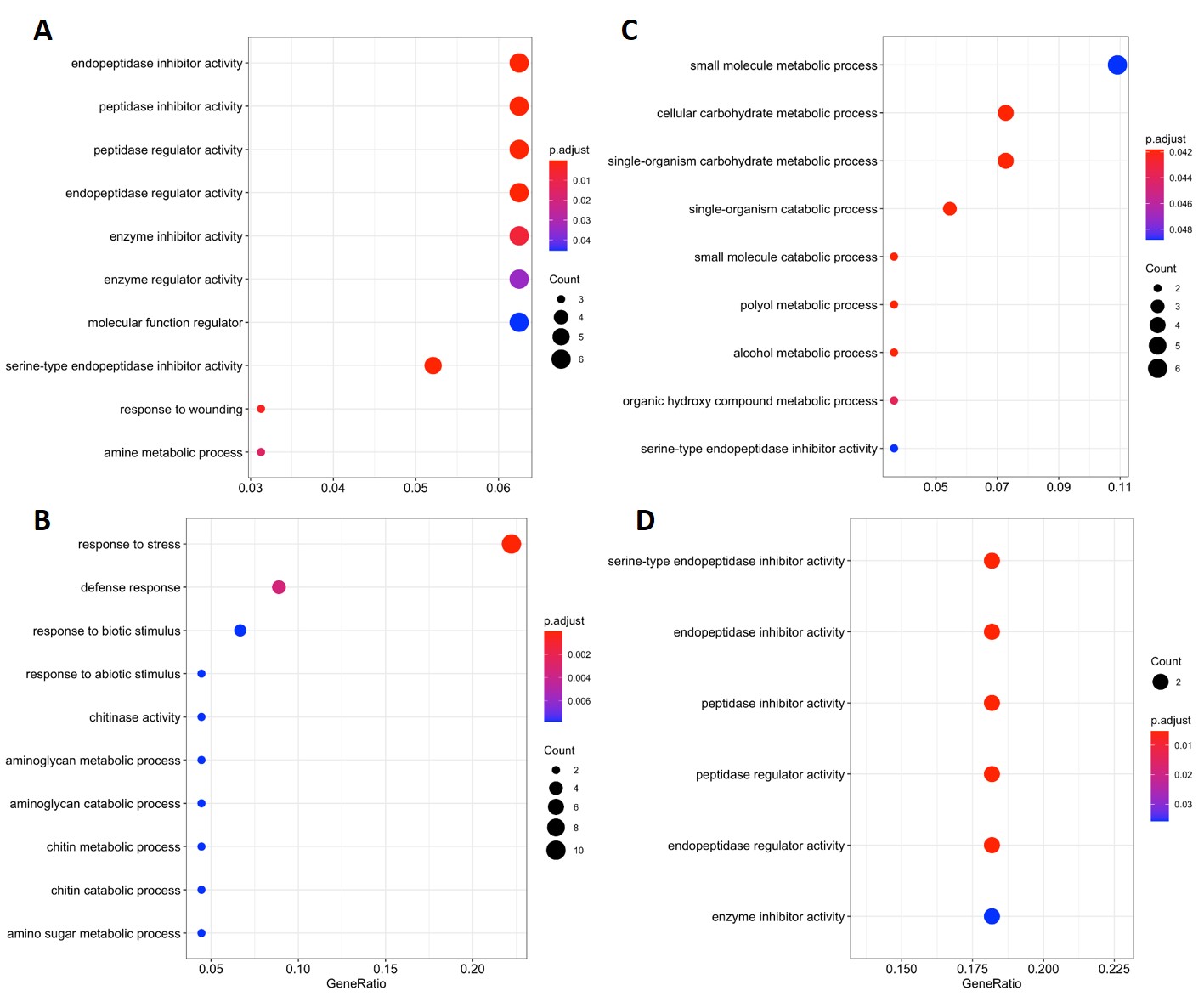

Supplement: Supplementary file 5 [file Image_3.JPEG]

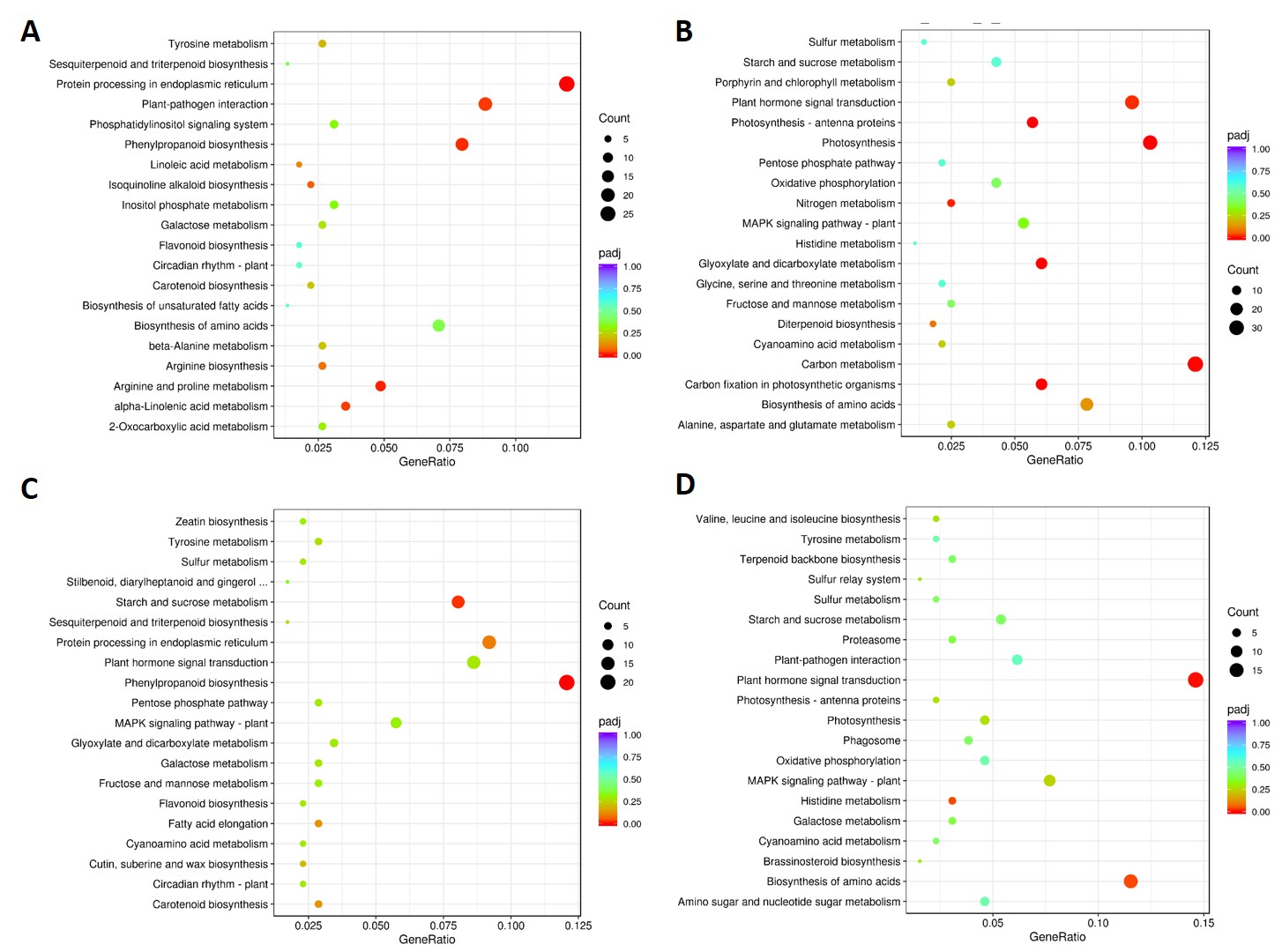

Supplement: Supplementary file 6 [file Image_4.JPEG]

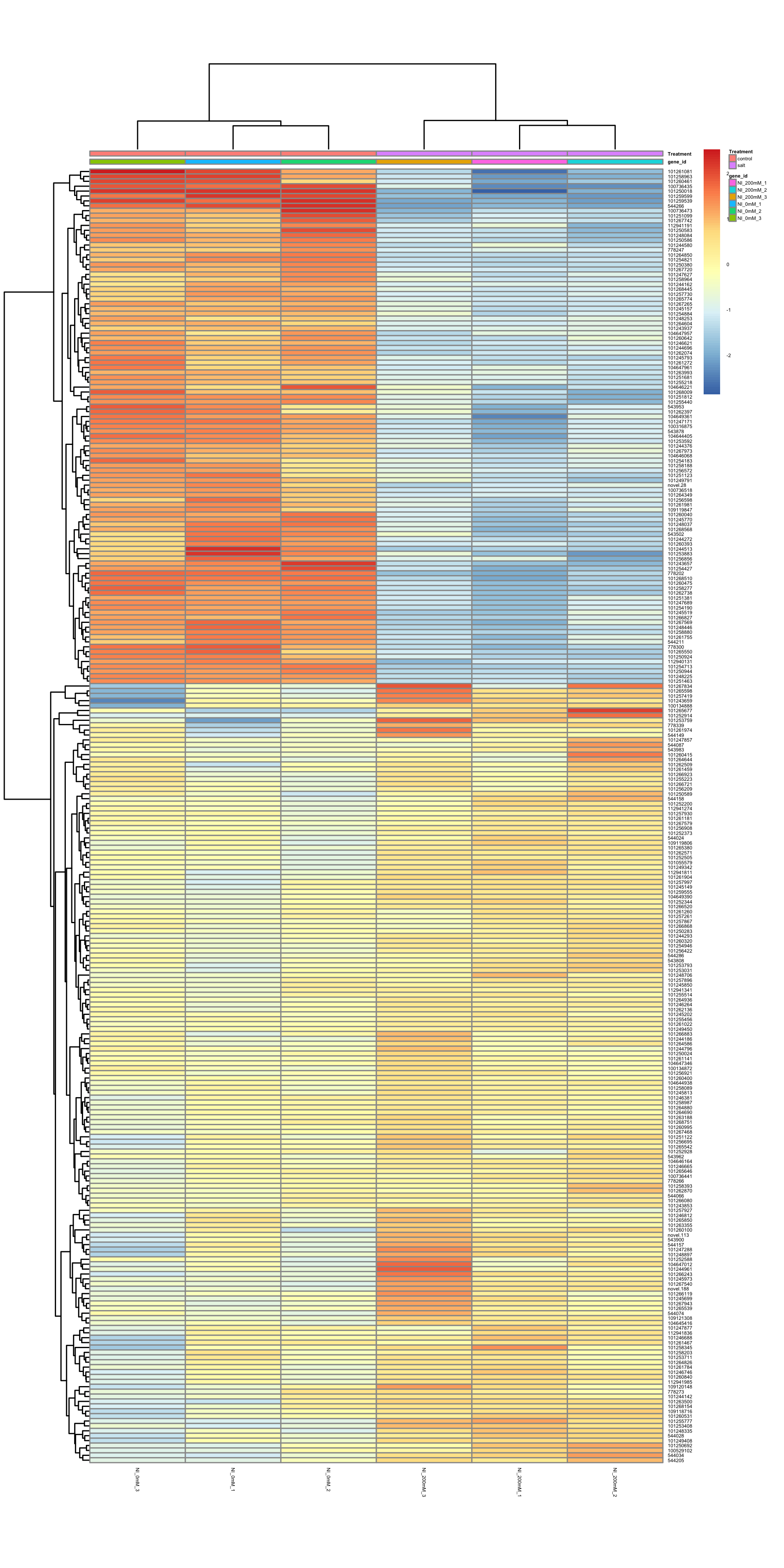

Supplement: Supplementary file 7 [file Image_5.PNG]

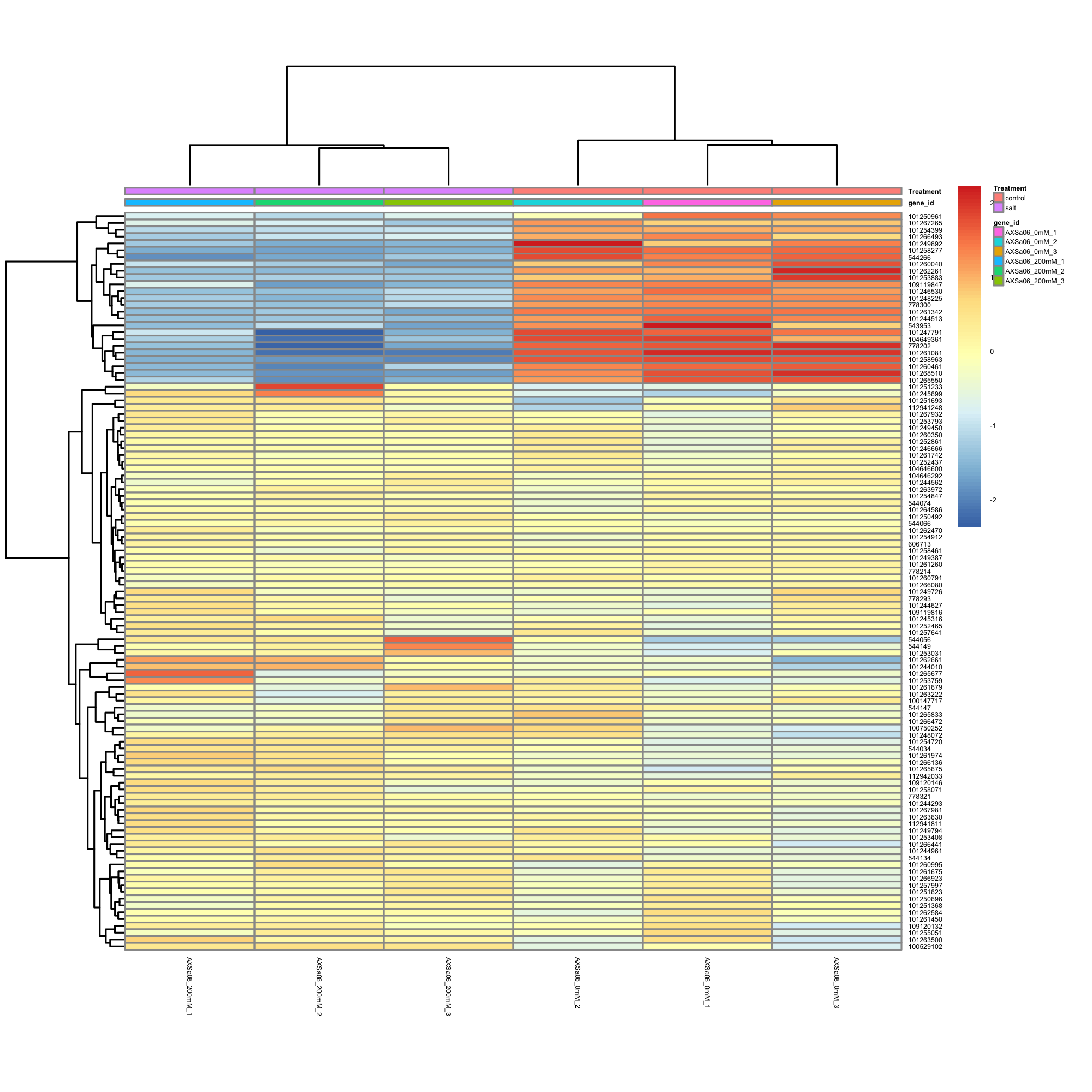

Supplement: Supplementary file 8 [file Image_6.PNG]

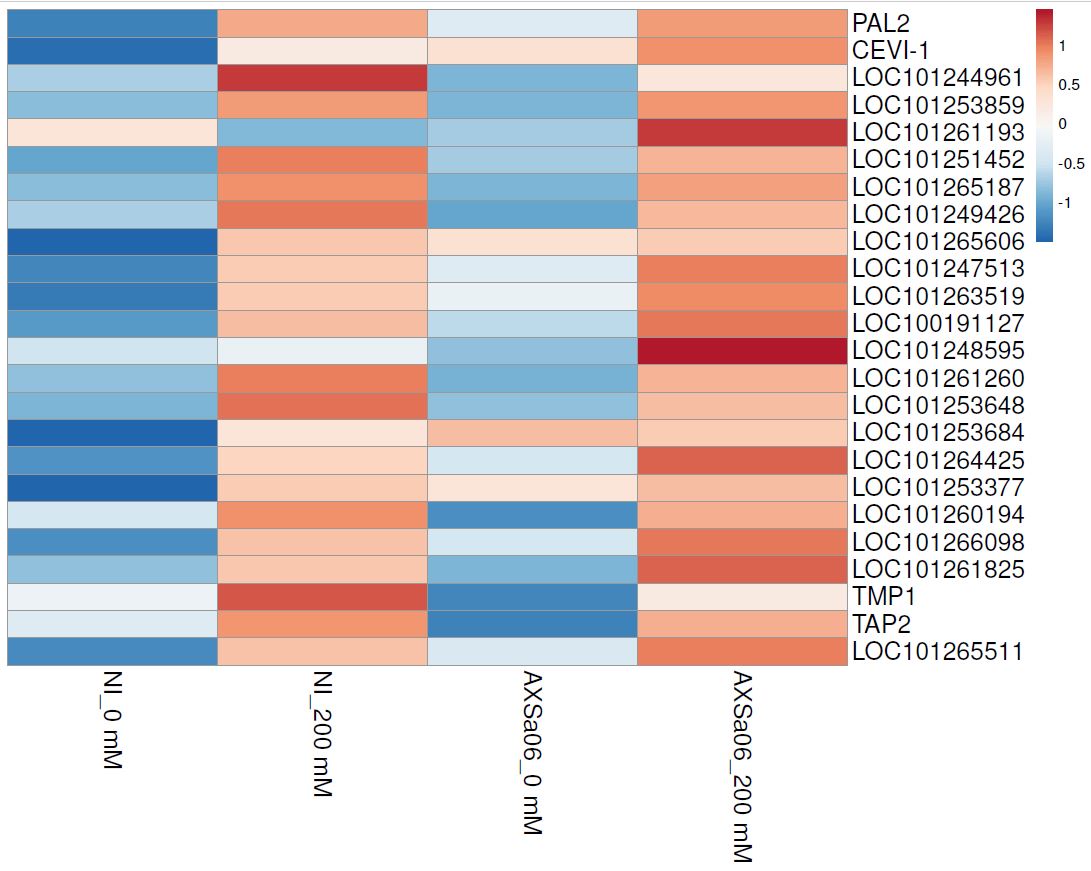

Supplement: Supplementary file 9 [file Image_7.JPEG]
